# Genome-Wide Identification, Sequence Variation, and Expression of the Glycerol-3-Phosphate Acyltransferase (GPAT) Gene Family in *Gossypium*

**DOI:** 10.3389/fgene.2019.00116

**Published:** 2019-02-20

**Authors:** Yupeng Cui, Jianjiang Ma, Guoyuan Liu, Nuohan Wang, Wenfeng Pei, Man Wu, Xingli Li, Jinfa Zhang, Jiwen Yu

**Affiliations:** ^1^State Key Laboratory of Cotton Biology, Cotton Institute of the Chinese Academy of Agricultural Sciences, Key Laboratory of Cotton Genetic Improvement, Ministry of Agriculture, Anyang, China; ^2^Department of Plant and Environmental Sciences, New Mexico State University, Las Cruces, NM, United States

**Keywords:** *Gossypium* spp., glycerol-3-phosphate acyltransferase (*GPAT*), gene expression patterns, abiotic stress, oil content

## Abstract

Cotton is an economically important crop grown for natural fiber and seed oil production. Cottonseed oil ranks third after soybean oil and colza oil in terms of edible oilseed tonnage worldwide. Glycerol-3-phosphate acyltransferase (*GPAT*) genes encode enzymes involved in triacylglycerol biosynthesis in plants. In the present study, 85 predicted *GPAT* genes were identified from the published genome data in *Gossypium*. Among them, 14, 16, 28, and 27 *GPAT* homologs were identified in *G. raimondii, G. arboreum, G. hirsutum*, and *G. barbadense*, respectively. Phylogenetic analysis revealed that a total of 108 *GPAT* genes from cotton, *Arabidopsis* and cacao could be classified into three groups. Furthermore, through comparison, the gene structure analyses indicated that *GPAT* genes from the same group were highly conserved between *Arabidopsis* and cotton. Segmental duplication could be the major driver for *GPAT* gene family expansion in the four cotton species above. Expression patterns of *GhGPAT* genes were diverse in different tissues. Most *GhGPAT* genes were induced or suppressed after salt or cold stress in Upland cotton. Eight *GhGPAT* genes were co-localized with oil and protein quantitative trait locus (QTL) regions. Thirty-two single nucleotide polymorphisms (SNPs) were detected from 12 *GhGPAT* genes, sixteen of which in nine *GhGPAT* genes were classified as synonymous, and sixteen SNPs in ten *GhGPAT* genes non-synonymous. Two SNP markers of the *GhGPAT16* and *GhGPAT26* genes were significantly correlated with cotton oil content in one of the three field tests. This study shed lights on the molecular evolutionary properties of *GPAT* genes in cotton, and provided reference for improvement of cotton response to abiotic stress and the genetic improvement of cotton oil content.

## Introduction

The majority of fatty acids are incorporated into membrane glycerolipids or triacylglycerol in a plant cells. The initial and committed step of glycerolipid biosynthesis is catalyzed by glycerol-3-phosphate acyltransferase, which catalyzes the transfer of a fatty acyl moiety from acyl-CoA, dicarboxylic acid-CoA or acyl-acyl carrier protein to the sn-1 or sn-2 position of glycerol 3-phosphate, resulting in the formation of lysophosphatic acid (LPA) (Zheng et al., 2003; Singer et al., 2016; Waschburger et al., 2018). There are plastid, endoplasmic reticulum (ER) and mitochondrial GPATs in plants (Gidda et al., 2009; Chen et al., 2011). Plastid GPATs participate in the prokaryotic biosynthesis of phospholipids, sulfolipids, and galactolipids (Ohlrogge and Browse, 1995), ER and mitochondrial GPATs are mainly involved in the synthesis of cutin, suberin, and storage lipids (Yang et al., 2012). The alignment of GPAT amino acid sequences from evolutionarily diverse organisms has revealed that all GPATs contain at least four highly conserved motifs, which are essential for both acyltransferase activity and the binding of glycerol-3-phosphate substrate (Lewin et al., 1999). The *GPAT* genes have been identified by genome-wide analyses in *Arabidopsis*, mammals and other plant species (Zheng et al., 2003; Gidda et al., 2009; Bertolesi et al., 2012; Yang et al., 2012; Waschburger et al., 2018), and fall within three different classes. Previous studies showed that *GPAT* gene is related to the fertility, oil content of plant, seed development and stress tolerance (Liu et al., 2013; Payá-Milans et al., 2016). In *Arabidopsis*, ten *GPAT* genes have been identified (Zheng et al., 2003; Gidda et al., 2009). *AtATS1* functions in the production of major phospholipids (Singer et al., 2016). *AtGPAT1* plays an essential role in pollen fertility, the disruption of *AtGPAT1* affects tapetal differentiation and causes most microspores to abort before reaching maturity, and *atgpat1*/*atgpat6* double mutants reduced the seed setting rate of *Arabidopsis* (Zheng et al., 2003). *AtGPAT4, AtGPAT5*, and *AtGPAT8* are involved in the production of cutin or suberin, *atgpat5* mutant could decrease suberin content in seed coat and root of *Arabidopsis* (Beisson et al., 2007), *atgpat4/atgpat8* double mutants could reduce cutin biosynthesis in *Arabidopsis*, while overexpression of *AtGPAT4 or AtGPAT8* in *Arabidopsis* could increase the content of C16 and C18 cutin monomers in leaves and stems by 80% (Li et al., 2007). *AtGPAT9* is known to be essential for seed triacylglycerol (TAG) biosynthesis (Gidda et al., 2009), knockdown of *AtGPAT9* can reduce seed oil content and an *atgpat9* mutant causes both male and female gametophytic lethality phenotypes (Shockey et al., 2016). Furthermore, *AtGPAT9* plays a role in the biosynthesis of polar lipids and TAG in developing leaves, as well as lipid droplet production in developing pollen grains of *Arabidopsis* (Singer et al., 2016).

Since the first identified plant *GPAT* gene was reported to function in tapetum differentiation and male fertility in *Arabidopsis* (Zheng et al., 2003), many studies have reported that *GPAT* genes are involved in adverse environmental conditions, such as cold, salt, and heat stress, indicating their potential roles in tolerance to abiotic stresses. The overexpression of the *AtATS1* gene has been reported to improve the tolerance of tobacco seedlings to chilling stress, while the transformation of a squash *GPAT* gene into tobacco resulted in decreased chilling tolerance (Murata and Tasaka, 1992). In rice, the overexpression of *AtATS1* gene increased the unsaturation of fatty acids in phosphatidylglycerol (PG) and improved the photosynthetic rates and growth at low temperatures (Yokoi et al., 1998; Ariizumi et al., 2002). The chloroplast *GPAT* gene from tomato (*LeGPAT)* was enhanced by chilling temperature (4°C) and suppressed by high temperature (40°C) in tomato leaves, and overexpression of the *LeGPAT* enhanced chilling tolerance by increasing the content of unsaturated fatty acids in PG (Sui et al., 2007b). Similarly, the overexpression of chloroplast *LeGPAT* in tomato enhanced chilling (Sui et al., 2007a) and salt tolerance (Sun et al., 2010), and the overexpression of Suaeda salsa *GPAT* gene *(SsGPAT)* in *Arabidopsis* enhanced salt tolerance (Sui et al., 2017). However, antisense-mediated depletion of chloroplast *LeGPAT* gene alleviated heat stress damage in transgenic tomato plants (Sui et al., 2010). Many other similar studies on *GPAT* genes and their functions have been reported (Payá-Milans et al., 2016; Misra et al., 2017; Xie et al., 2018). However, little is known about this gene family in cotton, especially their potential roles in lipid or oil synthesis or under salt and cold stress in Upland cotton.

Cotton (*Gossypium*) is the most important natural fiber-producing plant species for the textile industry, and according to the Foreign Agricultural Service, cotton is also the fifth largest vegetable oil-producing crop after soybeans, rapeseed, sunflower and peanuts in the world (https://apps.fas.usda.gov/psdonline/circulars/oilseeds.pdf). There are approximately 50 species of *Gossypium* including 45 diploid (2n = 2x = 26) and 5 tetraploid (2n = 4x = 52) species. Approximately 1–2 million years ago, diploid cotton species resembling *G. arboreum* (A2) and *G. raimondii* (D5) hybridized and underwent polyploidization, giving rise to tetraploid cotton species (Cronn et al., 1999; Paterson et al., 2012; Li et al., 2014). The recent progress in the whole-genome sequencing in *G. raimondii* (Paterson et al., 2012; Wang et al., 2012), *G. arboreum* (Li et al., 2014), *G. hirsutum* (Li et al., 2015; Zhang T. et al., 2015), and *G. barbadense* (Liu et al., 2015; Yuan et al., 2015) has rendered it possible to genome-wide analyses of important gene families including the *GPAT* gene family in the four species with their genomes sequenced.

In this study, all of the *GPAT* family genes in the four sequenced cotton species were identified and characterized. We further analyzed their phylogenetic tree, gene structure, chromosomal localization, functional diversification and expression profiles of *GPAT* genes in various tissues and under salt or cold stress. The duplication events of *GPAT* genes in the four cotton species were also analyzed. Finally, single nucleotide polymorphisms (SNPs) of *GhGPAT* genes were identified to study their association with cottonseed oil content using high-resolution melting (HRM). The results will contribute to widen our understanding about the gene structures and functions of *GPAT* family genes in *Gossypium*, and provide useful information on the relationship between *GhGPAT* genes and seed oil content in cotton.

## Materials and Methods

### Sequence Retrial and Identification of GPAT Family Members in *Gossypium*

The completed genome sequences of *G. raimondii* (Paterson et al., 2012), *G. arboreum* (Li et al., 2014), *G. hirsutum* acc. TM-1 (Zhang T. et al., 2015), and *G. barbadense* acc. 3-79 (Yuan et al., 2015) were downloaded from the CottonGen website1 (Yu et al., 2014). The genome sequences and the published GPAT proteins of *Arabidopsis* and *cacao* (*Theobroma cacao*) (Table S1) were retrieved from The *Arabidopsis* Information Resource (TAIR release 10, http://www.arabidopsis.org) and The Cacao Genome Database (https://www.cacaogenomedb.org), respectively. Afterwards, BlastP and tBlastN programs (*E*-value = 0.00001) were performed to search for the candidate *GPAT* genes of the four *Gossypium* species using GPAT protein sequences from *Arabidopsis* and *cacao* as queries. Subsequently, the Pfam (http://pfam.sanger.ac.uk/) (Finn et al., 2014), Interproscan (http://www.ebi.ac.uk/Tools/pfa/iprscan/) (Quevillon et al., 2005) and SMART (http://smart.embl-heidelberg.de/) (Letunic et al., 2015) databases were applied to confirm each candidate member of the GPAT family with the PlsC acyltransferase domain of Pfam (PF01553). Furthermore, the theoretical molecular weight (MW) and isoelectric point (*pI*) of the GPAT proteins were predicted using the online ExPASy tool (http://web.expasy.org/) (Bjellqvist et al., 1994), and subcellular localization was predicted using the online CELLO v2.5 server (http://cello.life.nctu.edu.tw/; Yu et al., 2004).

### Phylogenetic Construction

Clustal X v2.0 program (Larkin et al., 2007) was used to perform the multiple sequence alignment with default parameters, followed by manual comparisons and refinements. Subsequently, the Neighbor-Joining (NJ) method was used to construct different phylogenetic trees by MEGA v5.0 software (Tamura et al., 2011) with pairwise deletion option, poisson correction model and uniform rates. Bootstrap test were carried out with 1,000 replicates for evaluating the statistical reliability of phylogenetic tree. Furthermore, Maximum likelihood method was also applied in the tree construction to confirm the consistency with the NJ method.

### Chromosomal Localization, Gene Duplication and Genomic Organizations Prediction

All *GPAT* genes of *G. raimondii, G. arboreum, G. hirsutum*, and *G. barbadense* were mapped on the corresponding chromosomes according to their positional information provided in the genome annotation document. The chromosome location images of cotton *GPAT* genes were portrayed by Mapchart v2.2 software (Voorrips, 2002) and Circos tool (Krzywinski et al., 2009). *GPAT* gene duplication events were defined according to the length of aligned sequence covered more than 80% of the longer gene, similarity of the aligned regions was bigger than 80%, and only one duplication event was counted for tightly linked genes (Maher et al., 2006; Dong et al., 2016; Cui et al., 2017). Referring to the chromosomal locations of *GPAT* genes, these *GPAT* genes could be designated as segmental duplications or tandem duplications.

The Gene Structure Display Server (GSDS) tool (Hu et al., 2015) was used to construct the gene structure of *GPAT* genes by comparing the predicted *GPAT* coding sequences with their corresponding genomic sequences.

### Selective Pressure Analysis of Duplicated Gene Pairs

All the full-length gene sequence of the *GPAT* duplicated gene pairs of *G. raimondii, G. arboreum, G. hirsutum*, and *G. barbadense* were firstly aligned by Clustal X v2.0 program, and then the synonymous substitution (Ks) and non-synonymous substitution (Ka) were calculated using the DnaSP v5.0 software (Rozas et al., 2003). Finally, the Ka/Ks ratio was used to estimate the selection pressure for each gene pair.

### RNA-Seq Data Analysis and Gene Expression Heatmap

The raw RNA-Seq data of *G. hirsutum* acc. TM-1 was downloaded from the NCBI under the accession number SRA: PRJNA248163 (Zhang T. et al., 2015). TopHat and cufflinks were used for mapping reads and analyzing gene expression levels, and fragments per kilobase million (FPKM) values were used to normalize gene expression levels (Trapnell et al., 2013). The expression level of *GhGPAT* family genes at different developmental stages were measured by extracting their respective data from the total expression matrix according to the identified *GhGPAT* ID. The expression levels of ten *AtGPAT* genes were extracted form publicly *Arabidopsis* microarray expression datasets by the BioArray Resource (BAR) Expression Profiler tool (http://bar.utoronto.ca/affydb/cgi-bin/affy_db_exprss_browser_in.cgi) (Toufighi et al., 2005). The heatmap for expression profiles were generated with MultiExperiment Viewer (MeV) software.

### Cis-Acting Elements Searching in the Promoters of GhGPATs

To identify the *cis*-elements in the promoter sequences of the 28 *GPAT* family genes in *G. hirsutum*, the BLASTN program was applied for searching *G. hirsutum* genome using the *GhGPAT* genes as query sequences. A 2-kb of genomic sequences upstream of the start codon of each *GhGPAT* gene was chosen, and then each sequence was submitted to the PlantCARE database (Lescot et al., 2002) (http://bioinformatics.psb.ugent.be/webtools/plantcare/html/) to identify the *cis*-elements.

### Plant Materials and Abiotic Stress Treatment

Cotton seedlings of *G. hirsutum* L. acc TM-1 were grown in a temperature-controlled chamber at 28 ± 2°C under 16 h light/ 8 h dark condition. Seedlings at the 3-true leaf stage were exposed to the following different treatments. For low temperature, seedlings were grown in a temperature-controlled chamber at 4°C for 0, 12, and 24 h. For salt treatment, the seedlings were grown in Hoagland nutrient solution with additional 0, 150, 200, and 300 mM NaCl for 24 h, which represented the control condition, slight stress, moderate stress, and severe stress, respectively. After different treatments, the roots, stems, cotyledons and leaves were harvested at each time point of each treatment. Three biological replicates were conducted for each time point. All collected samples were immediately frozen with liquid nitrogen and then stored at −80°C for RNA isolation.

### Quantitative Reverse Transcriptase-Polymerase Chain Reaction (qRT-PCR) Analysis

Total RNA of all samples was extracted using an RNAprep Pure Plant kit (Tiangen, Beijing, China), and approximately 1 μg RNA was used for the synthesis of first-strand cDNAs with PrimerScript 1st Strand cDNA synthesis kit (TaKaRa, Dalian, China) according to the manufacturer protocol. The gene-specific primers were designed, according to the CDSs of cotton *GPAT* genes, using Primer v5.0 software. The specific primers used are listed in Table S2, and *GhUBQ7* was used as an internal control to normalize all data. The qRT-PCR was strictly conducted with SYBR premix Ex Taq Kit (TakaRa) following the manufacturer's instruction using ABI 7500 real-time PCR System (Applied Biosystems, Foster City, CA, USA). Each sample was conducted with three biological replicates and three technical replicates. The relative expression levels of *GhGPAT* genes were calculated using the 2^−ΔΔCt^ method (Livak and Schmittgen, 2001).

### Identification of Single Nucleotide Polymorphisms (SNPs) for GPAT Genes and Statistical Analysis With Cottonseed Oil

Single-nucleotide polymorphism (SNPs) for *GPAT* genes were identified by the RNA-seq datasets of CRI 36 and Hai 7124, in which CRI 36 is a currently commercial cultivar of *G. hirsutum* and Hai 7124 is a non-commercial *G. barbadence*, and SOAPsnp software was used to scan SNPs based on the assembled contigs (Li et al., 2009). To validate SNP markers, genomic DNA was extracted from an interspecific backcross inbred line (BIL) population of 180 lines, as well as the two parents using a quick CTAB method (Zhang and Stewart, 2000). In this study, the BIL population was developed from a cross between Upland cotton CRI 36 and *G. barbadense* Hai 7124 through one generation of backcrossing using CRI 36 as the recurrent parent followed by seven generations of selfing. Putative SNPs were identified to design primers using Primer v5.0 software (Table S2) and analyzed their association with cottonseed oil content using high-resolution melting (HRM). The HRM reaction mixture and PCR followed the protocols (Ma et al., 2016). The SNP markers were coded as “1” for genotypes of CRI 36, “3” for genotypes of Hai 7124 and “2” for the heterozygotes, and used for a simple correlation analysis with the cottonseed oil content in the BIL population by SPPS software (IBM, New York, USA).

## Results

### Genome-Wide Identification of GPAT Genes in Four Cotton Species

To identify all the *GPAT* genes in cotton, we performed a BLASTP search against the diploid cotton (*G. raimondii* and *G. arboreum*) and tetraploid cotton (*G. hirsutum* and *G. barbadense*) protein databases using the *GPAT* sequences of *Arabidopsis* (10) and *cacao* (13) as queries (Table S1). All potential cotton proteins were then subjected to domain analysis using the Pfam, Interproscan and SMART databases to further confirm the presence of the GPAT protein domain (PF01553). Eventually, a total of 14, 16, 28, and 27 *GPAT* members were identified in *G. raimondii, G. arboreum, G. hirsutum* and *G. barbadense*, respectively. These *GPAT* genes were named consecutively from *GrGPAT1*-*GrGPAT14, GaGPAT1*-*GaGPAT16, GhGPAT1*-*GaGPAT28*, and *GbGPAT1*-*GaGPAT27* in the four cotton species, respectively, according to the order of their chromosomal locations (Table 1). Eighty of the 85 identified *GPAT* genes encode proteins ranging between 377 and 543 amino acids (AAs), except for 5 genes with lengths of less than 377 AAs or more than 543 AAs (i.e., *GrGPAT3* encodes a protein of 261 AAs, and *GhGPAT20, GbGPAT2, GbGPAT16*, and *GbGPAT20* encode proteins of 258, 319, 740, and 298 AAs, respectively). The predicted Mw of the GPAT proteins in the four cotton species ranged from 28.78 to 83.20 kDa, and the theoretical *pI* varied between 5.49 and 9.63. The protein subcellular localization of GPAT in cotton showed that 68 of 85 GPAT proteins were predicted to be located in the plasma membrane, while the rest were predicted to be located in the chloroplast or cytoplasm. We further analyzed the orthologous and paralogous *GPAT* genes in the four cotton species (Table S3). We identified 18 orthologous gene pairs between *G. hirsutum* and *G. barbadense*. Compared with the *G. raimondii*, 13, 12, and 10 orthologous gene pairs were identified for *G. arboreum, G. hirsutum*, and *G. barbadense*, respectively. Compared with the *G. arboreum*, 10 and 9 orthologous gene pairs were identified for *G. hirsutum* and *G. barbadense*, respectively. In addition, 5, 6, 13 and 11 paralogous gene pairs were identified in *G. raimondii, G. arboreum, G. hirsutum*, and *G. barbadense*, respectively.

**Table 1:** The information of *GPAT* gene family in four *Gossypium* species.

**Gene name**	**Gene identifier**	**Genomics position**	**CDS**	**AA size**	**Mw (kDa)**	**pI**	**Predicted subcellular location**
*GrGPAT1*	Gorai.003G185500.1	Chr03: 45619381-45625850	1386	461	51.7	8.38	Chloroplast
*GrGPAT2*	Gorai.004G145100.1	Chr04: 40873197-40875543	1632	543	61.38	9.59	Plasma Membrane
*GrGPAT3*	Gorai.004G182200.1	Chr04: 49380119-49387779	786	261	29.21	6.66	Chloroplast
*GrGPAT4*	Gorai.005G258500.1	Chr05: 63451173-63455894	1134	377	43.16	7.96	Plasma Membrane
*GrGPAT5*	Gorai.006G181200.1	Chr06: 43856590-43859232	1515	504	56.1	9.31	Plasma Membrane
*GrGPAT6*	Gorai.007G033500.1	Chr07: 2286486-2290897	1155	384	42.86	5.52	Chloroplast
*GrGPAT7*	Gorai.007G100100.1	Chr07: 7410329-7415092	1503	500	55.74	9.29	Plasma Membrane
*GrGPAT8*	Gorai.008G098000.1	Chr08: 27391572-27393956	1626	541	61.01	9.26	Plasma Membrane
*GrGPAT9*	Gorai.010G045500.1	Chr10: 4807029-4809534	1551	516	58.4	9.56	Plasma Membrane
*GrGPAT10*	Gorai.011G267100.1	Chr11: 59897897-59899780	1485	494	55.27	9.05	Plasma Membrane
*GrGPAT11*	Gorai.011G267200.1	Chr11: 59914117-59915636	1218	405	45.57	9.04	Plasma Membrane
*GrGPAT12*	Gorai.012G071300.1	Chr12: 10531559-10533384	1467	488	54.9	9.54	Plasma Membrane
*GrGPAT13*	Gorai.012G112800.1	Chr12: 25762804-25765226	1548	515	57.38	9.28	Plasma Membrane
*GrGPAT14*	Gorai.012G158300.1	Chr12: 33150125-33154932	1137	378	43.41	8.83	Plasma Membrane
*GaGPAT1*	Cotton_A_09737	chr03: 35875883-35882520	1341	446	49.93	6.69	Chloroplast
*GaGPAT2*	Cotton_A_21450	chr04: 24644639-24648923	1467	488	54.25	9.26	Plasma Membrane
*GaGPAT3*	Cotton_A_20607	chr04: 56175430-56177058	1533	510	56.14	9.23	Plasma Membrane
*GaGPAT4*	Cotton_A_00592	chr05: 8890287-8894119	1134	377	43.25	8.83	Plasma Membrane
*GaGPAT5*	Cotton_A_18440	chr08: 44876559-44878590	1548	515	58.37	9.63	Plasma Membrane
*GaGPAT6*	Cotton_A_30920	chr09: 80514225-80515788	1476	491	54.87	9.12	Plasma Membrane
*GaGPAT7*	Cotton_A_24431	chr09: 85523484-85525099	1515	504	55.11	9.23	Plasma Membrane
*GaGPAT8*	Cotton_A_25400	chr10: 112459178-112464741	1386	461	51.92	8.1	Chloroplast
*GaGPAT9*	Cotton_A_02486	chr11: 20915572-20917911	1503	500	55.52	9.28	Plasma Membrane
*GaGPAT10*	Cotton_A_39465	chr12: 20976325-20978378	1506	501	55.86	9.26	Plasma Membrane
*GaGPAT11*	Cotton_A_19822	chr12: 45334367-45336407	1602	533	60.35	9.49	Plasma Membrane
*GaGPAT12*	Cotton_A_36957	chr12: 50316570-50318108	1467	488	54.79	9.45	Plasma Membrane
*GaGPAT13*	Cotton_A_25468	chr12: 75489147-75491122	1626	541	60.98	9.13	Plasma Membrane
*GaGPAT14*	Cotton_A_08860	chr12: 90634082-90638451	1137	378	43.41	8.83	Plasma Membrane
*GaGPAT15*	Cotton_A_09184	chr13: 44533469-44537359	1155	384	42.67	5.49	Cytoplasmic
*GaGPAT16*	Cotton_A_24308	chr13: 89011942-89013603	1578	525	59.13	9.24	Plasma Membrane
*GhGPAT1*	Gh_A03G1851	A03: 99577941-99581782	1134	377	43.24	8.6	Plasma Membrane
*GhGPAT2*	Gh_A04G0765	A04: 51891051-51893104	1506	501	55.82	9.26	Plasma Membrane
*GhGPAT3*	Gh_A04G1069	A04: 60750094-60754552	1137	378	43.41	8.83	Plasma Membrane
*GhGPAT4*	Gh_A05G3037	A05: 78024613-78026151	1467	488	54.79	9.51	Plasma Membrane
*GhGPAT5*	Gh_A06G0327	A06: 4927618-4929887	1566	521	59.02	9.63	Plasma Membrane
*GhGPAT6*	Gh_A08G1054	A08: 72959792-72961831	1599	532	60.17	9.43	Plasma Membrane
*GhGPAT7*	Gh_A08G1388	A08: 88694103-88700765	1341	446	49.8	6.52	Chloroplast
*GhGPAT8*	Gh_A08G1816	A08: 98752308-98754008	1617	538	60.72	9.27	Plasma Membrane
*GhGPAT9*	Gh_A09G1528	A09: 68529716-68532054	1503	500	55.56	9.28	Plasma Membrane
*GhGPAT10*	Gh_A10G1987	A10: 97626943-97628506	1476	491	54.88	9.12	Plasma Membrane
*GhGPAT11*	Gh_A11G0804	A11: 7991963-7996279	1503	500	55.83	9.25	Plasma Membrane
*GhGPAT12*	Gh_A12G0824	A12: 49192781-49194756	1626	541	60.99	9.13	Plasma Membrane
*GhGPAT13*	Gh_D02G2290	D02: 66653634-66657584	1134	377	43.16	7.96	Plasma Membrane
*GhGPAT14*	Gh_D03G1661	D03: 46629378-46634196	1386	461	51.69	8.1	Chloroplast
*GhGPAT15*	Gh_D04G0605	D04: 10821266-10822804	1467	488	54.93	9.48	Plasma Membrane
*GhGPAT16*	Gh_D04G1251	D04: 41151043-41153134	1548	515	57.41	9.28	Plasma Membrane
*GhGPAT17*	Gh_D04G1675	D04: 48923650-48927352	1086	361	41.48	9.02	Plasma Membrane
*GhGPAT18*	Gh_D06G0355	D06: 4862412-4864432	1548	515	58.23	9.54	Plasma Membrane
*GhGPAT19*	Gh_D08G1335	D08: 43709349-43711332	1632	543	61.41	9.58	Plasma Membrane
*GhGPAT20*	Gh_D08G1336	D08: 43711996-43712772	777	258	28.78	9.35	Plasma Membrane
*GhGPAT21*	Gh_D08G1683	D08: 52638770-52645877	1341	446	49.86	6.48	Chloroplast
*GhGPAT22*	Gh_D09G1546	D09: 43059728-43061991	1509	502	55.89	9.23	Plasma Membrane
*GhGPAT23*	Gh_D10G2294	D10: 61148398-61149971	1485	494	55.27	9.05	Plasma Membrane
*GhGPAT24*	Gh_D11G0942	D11: 8173864-8178049	1503	500	55.74	9.29	Plasma Membrane
*GhGPAT25*	Gh_D12G0839	D12: 26672950-26674926	1626	541	61	9.22	Plasma Membrane
*GhGPAT26*	Gh_A03G1948	scaffold496_A03: 45464-51022	1386	461	51.9	8.1	Chloroplast
*GhGPAT27*	Gh_A11G2987	scaffold2726_A11: 85575-89619	1323	440	48.69	6.11	Chloroplast
*GhGPAT28*	Gh_D11G3539	scaffold4571_D11: 33114-37148	1323	440	49.01	6.06	Chloroplast
*GbGPAT1*	Gbscaffold10683.8.0	A03: 101375090-101379793	1134	377	43.24	8.6	Plasma Membrane
*GbGPAT2*	Gbscaffold9797.1.0	A04: 63446426-63449956	960	319	36.48	8.48	Plasma Membrane
*GbGPAT3*	Gbscaffold1022.17.0	A06: 5684360-5686814	1548	515	58.41	9.63	Plasma Membrane
*GbGPAT4*	Gbscaffold127.8.0	A08: 76313638-76315690	1599	532	60.17	9.43	Plasma Membrane
*GbGPAT5*	Gbscaffold5413.16.0	A08: 103987721-103989621	1617	538	60.69	9.19	Plasma Membrane
*GbGPAT6*	Gbscaffold31407.2.0	A09: 71102212-71105277	1503	500	55.61	9.28	Plasma Membrane
*GbGPAT7*	Gbscaffold5583.14.0	A10: 103589265-103590767	1194	397	44.35	9.44	Plasma Membrane
*GbGPAT8*	Gbscaffold17219.12.0	A11: 9067020-9071621	1503	500	55.86	9.25	Plasma Membrane
*GbGPAT9*	Gbscaffold6816.2.0	A12: 51866337-51868435	1626	541	60.96	9.17	Plasma Membrane
*GbGPAT10*	Gbscaffold17185.12.0	D02: 70322327-70329230	1134	377	43.18	8.23	Plasma Membrane
*GbGPAT11*	Gbscaffold2076.5.0	D04: 50119564-50124142	1137	378	43.43	8.83	Plasma Membrane
*GbGPAT12*	Gbscaffold2076.7.0	D04: 50128492-50133233	1137	378	43.43	8.83	Plasma Membrane
*GbGPAT13*	Gbscaffold11060.1.0	D06: 4727967-4730037	1548	515	58.38	9.57	Plasma Membrane
*GbGPAT14*	Gbscaffold5633.11.0	D08: 44543424-44545643	1632	543	61.31	9.55	Plasma Membrane
*GbGPAT15*	Gbscaffold15529.6.0	D08: 54558468-54565707	1341	446	49.86	6.34	Chloroplast
*GbGPAT16*	Gbscaffold5633.10.0	D08: 44527931-44530917	2223	740	83.2	9.42	Plasma Membrane
*GbGPAT17*	Gbscaffold7965.5.0	D09: 45190899-45193553	1509	502	55.91	9.23	Plasma Membrane
*GbGPAT18*	Gbscaffold10055.26.0	D10: 62642543-62644388	1485	494	55.27	9.12	Plasma Membrane
*GbGPAT19*	Gbscaffold1641.10.0	D11: 9489598-9494108	1503	500	55.77	9.29	Plasma Membrane
*GbGPAT20*	Gbscaffold346.41.0	D11: 3136620-3154735	897	298	33.65	5.64	Cytoplasmic
*GbGPAT21*	Gbscaffold3997.11.0	D12: 26167138-26169961	1626	541	60.98	9.26	Plasma Membrane
*GbGPAT22*	Gbscaffold1135.1.0	scaffold1135: 10610-20038	1341	446	49.8	6.52	Chloroplast
*GbGPAT23*	Gbscaffold14639.21.0	scaffold14639_A03: 268323-274714	1692	563	64.22	9.38	Chloroplast
*GbGPAT24*	Gbscaffold16283.3.0	scaffold16283: 71319-73133	1467	488	54.93	9.48	Plasma Membrane
*GbGPAT25*	Gbscaffold3695.4.0	scaffold3695: 56371-64196	1386	461	51.69	8.37	Chloroplast
*GbGPAT26*	Gbscaffold9785.1.0	scaffold9785: 126689-128615	1383	460	51.37	9.35	Plasma Membrane
*GbGPAT27*	Gbscaffold9785.2.0	scaffold9785: 129516-131971	1548	515	57.3	9.23	Plasma Membrane

### Phylogenetic Analysis of the GPAT Gene Family

**Figure 1:**
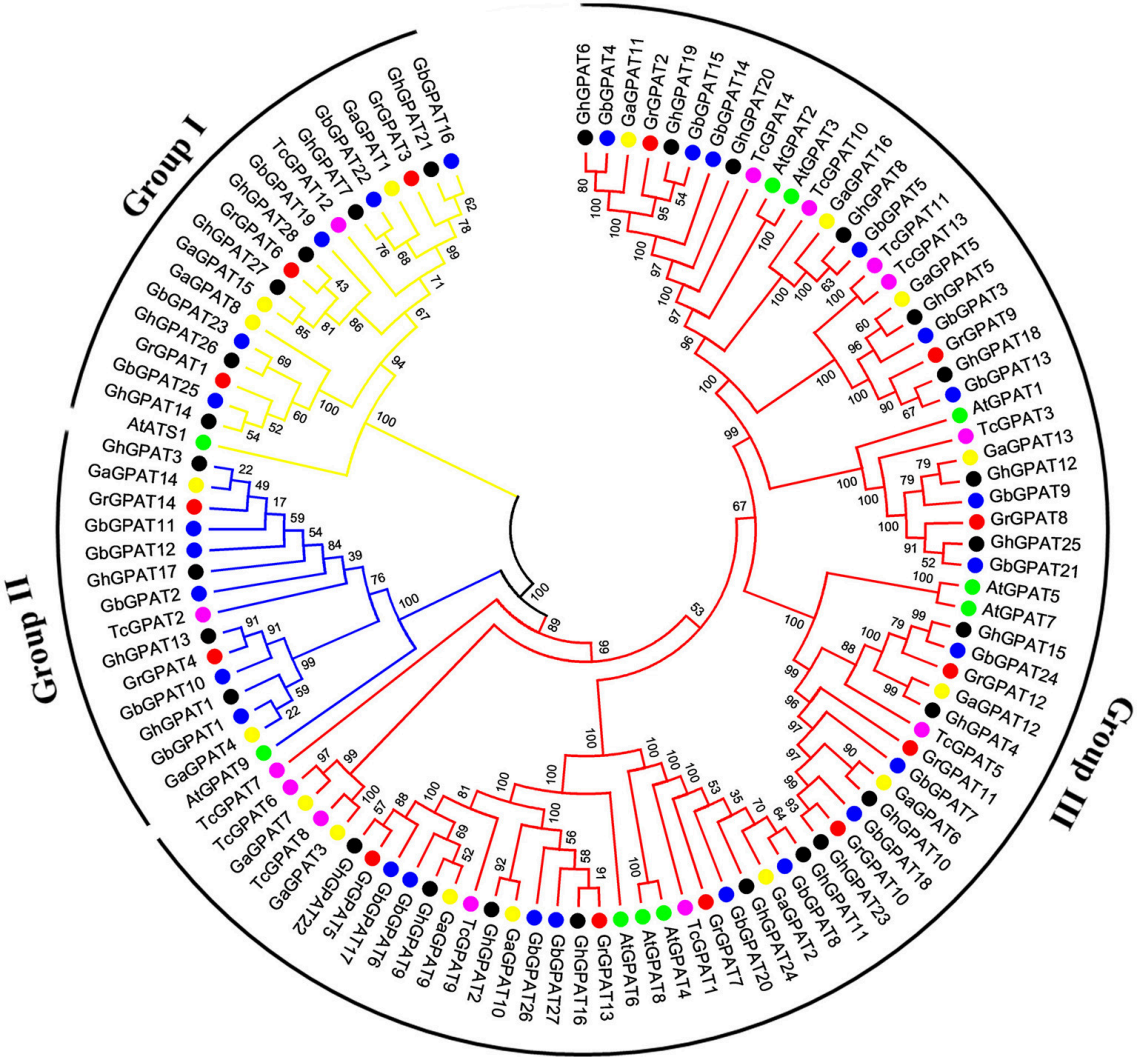
Phylogenetic relationships of *GPAT* genes from *Gossypium, Arabidopsis*, and *cacao*. Phylogenetic analysis was performed using the neighbor-joining method with 1,000 replicates. The *GPAT* genes from *G. raimondii, G. arboreum, G. hirsutum, G. barbadense, Arabidopsis*, and cacao were marked with the red, yellow, black, blue, green, and magenta dots, respectively. The branches of each group were indicated in a specific color.

To assess the phylogenetic relationships of *GPAT* genes among the four cotton species (*G. raimondii, G. arboreum, G. hirsutum*, and *G. barbadense*) and other species, a comprehensive phylogenetic tree was constructed using a NJ method (Figure 1). Meanwhile, the phylogenetic tree was reconstructed with Maximum likelihood (ML) method, which was almost identical with only minor difference at some branches (Figure S1), suggesting that the two methods were largely consistent with each other. As shown in Figure 1, *GPAT* genes could be divided into three groups, named the Group I, Group II, and Group III. Group III has the most members (74), followed by Group I (19), and Group II (15). Compared to *Arabidopsis, GPAT* genes in the four cotton species showed a closer relationship with those from cacao, since they were clustered closely to each other in the phylogenetic tree. However, the number of *GPAT* genes in cotton and cacao were not similar within the groups, and one cacao gene corresponding to one to three homologous genes of *G. raimondii* and *G. arboreum*. For example, in Group I, *TcGPAT12* had three orthlogous in *G. raimondii* and *G. arboreum* each, whereas in Group II, *TcGPAT2* had two orthologous genes in both *G. raimondii* and *G. arboreum*. Furthermore, in Group III, *TcGPAT4* had only one orthologous gene in *G. raimondii* and *G. arboreum*, respectively. In addition, the *GPAT* genes from the four cotton species showed a closer relationship than that from *Arabidopsis* and *cacao* (Figure 1). From the perspective of evolution, each *GPAT* gene in *G. raimondii* corresponds to one orthologous gene in *G. arboreum* and to two orthologous genes in tetraploid *G. hirsutum* and *G. barbadense*, since the diploid genomes of *G. raimondii* and *G. arboreum* are the progenitors for tetraploid *G. hirsutum* and *G. barbadense* (Paterson et al., 2012). In this study, most *GPAT* genes from *G. hirsutum* and from *G. barbadense* showed a one-to-one correspondence with those from the diploid progenitors (*G. raimondii* and *G. arboreum*). The inconsistencies were that the *GrGPAT2* gene was duplicated in *G. arboreum, G. hirsutum*, and *G. barbadense*, implying that this gene underwent an independent duplication event in *G. arboreum* after its divergence from *G. raimondii*, even this duplication also existed after the formation of tetraploid cotton. In addition, *GPAT* genes from A subgenome and D subgenome of *G. hirsutum* showed a bias to those from *G. arboreum* and *G. raimondii*.

### Genomic Structure of GPAT Genes

**Figure 2:**
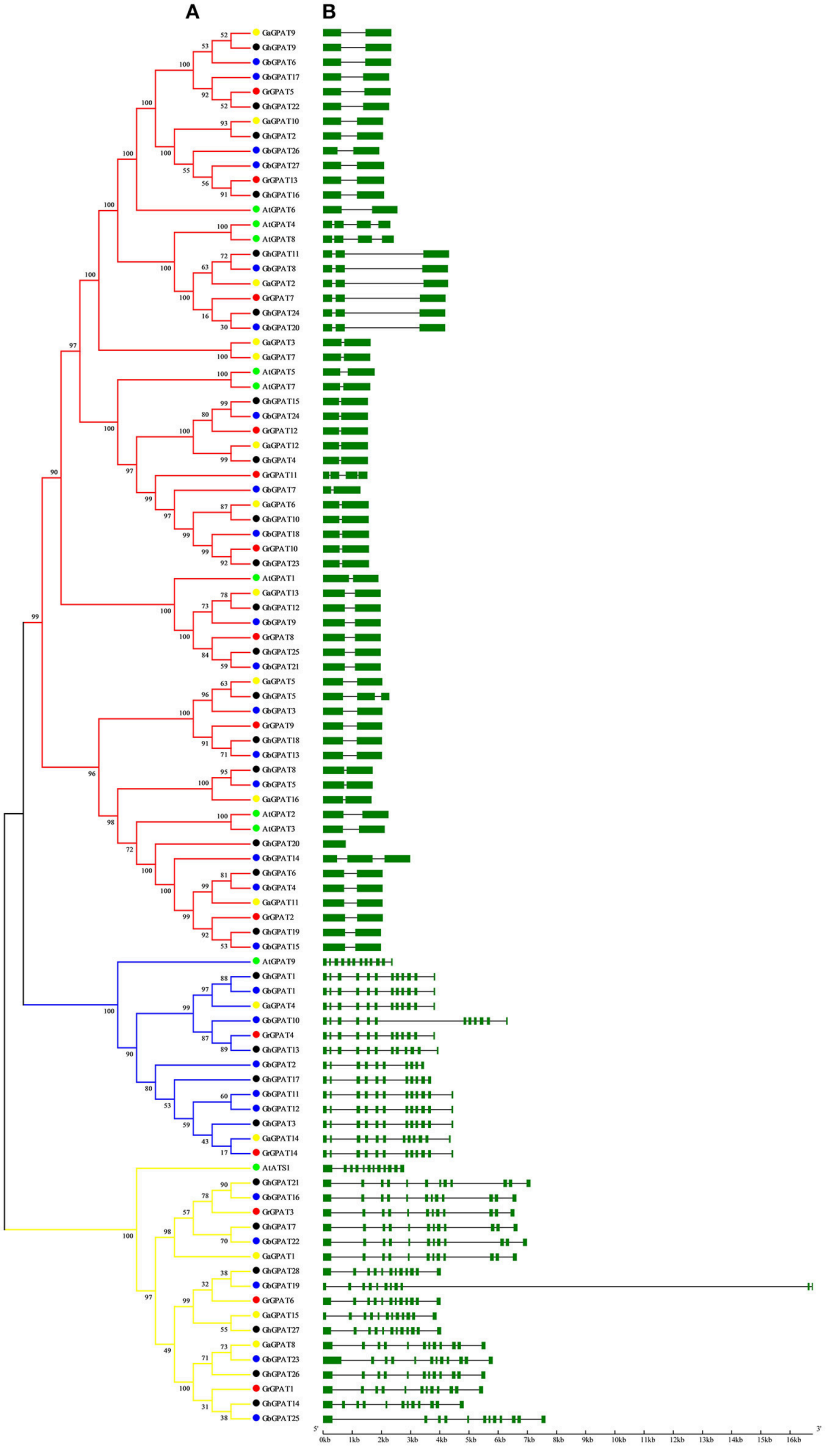
Phylogenetic relationships and gene structure of the *GPAT* gene family in *Gossypium* and *Arabidopsis*. **(A)** The phylogenetic tree of all GPAT proteins in *G. raimondii, G. arboreum, G. hirsutum, G. barbadense*, and *Arabidopsis* was constructed by the neighbor-joining method with 1,000 replicates, **(B)** The exon-intron structure of *GPAT* genes in *G. raimondii, G. arboreum, G. hirsutum, G. barbadense*, and *Arabidopsis*. Exons are represented by green boxes and introns by black lines.

Gene structure of *GPAT* genes is closely related to its function, and could reflect the phylogenetic relation among the *GPAT* genes, together with their phylogenetic analysis. To obtain further insight into the evolutionary relationships among *GPAT* genes from the four cotton species and *Arabidopsis*, we compared their gene structures. As shown in Figure 2A, the *GPAT* genes were divided into three groups as described in Figure 1. The *GPAT* gene members within groups were mostly the same, although the topologies of the two phylogenetic trees were different. Analysis of *Arabidopsis* and cotton *GPAT* gene structure for exon/intron organizations revealed that the number of exons per gene varied from 1 to 12 (Figure 2B), and the genes close to each other in the phylogenetic tree showed highly similar exon numbers. In general, the *GPAT* genes in the same group had similar exons than other groups. For example, all the members in Group I contained 12 exons except *GbGPAT19* that had 11 exons. A host of *GPAT* genes in Group II possessed 12 exons, except for *GhGPAT17* and *GbGPAT2*, which had 11 and 10 exons, respectively. Most members of Group III had two exons, except for *GhGPAT20* that had only one exon, eight genes (*GaGPAT2, GrGPAT7, GhGPAT5, GhGPAT11, GhGPAT24, GbGPAT8, GbGPAT14*, and *GbGPAT20*) that had three exons, and three enes (*AtGPAT4, AtGPAT8*, and *GrGPAT11*) that had four exons. As excepted, the gene structures of orthologous gene pairs of cotton *GPATs* were almost identical with minor differences with the exception of *GrGPAT6*/*GbGPAT19, GaGPAT14*/*GbGPAT2, GaGPAT15*/*GbGPAT19, GaGPAT5*/*GhGPAT5*, and *GhGPAT5*/*GbGPAT3* (Table S3).

### Genomic Localization and Duplication of GPAT Genes

**Figure 3:**
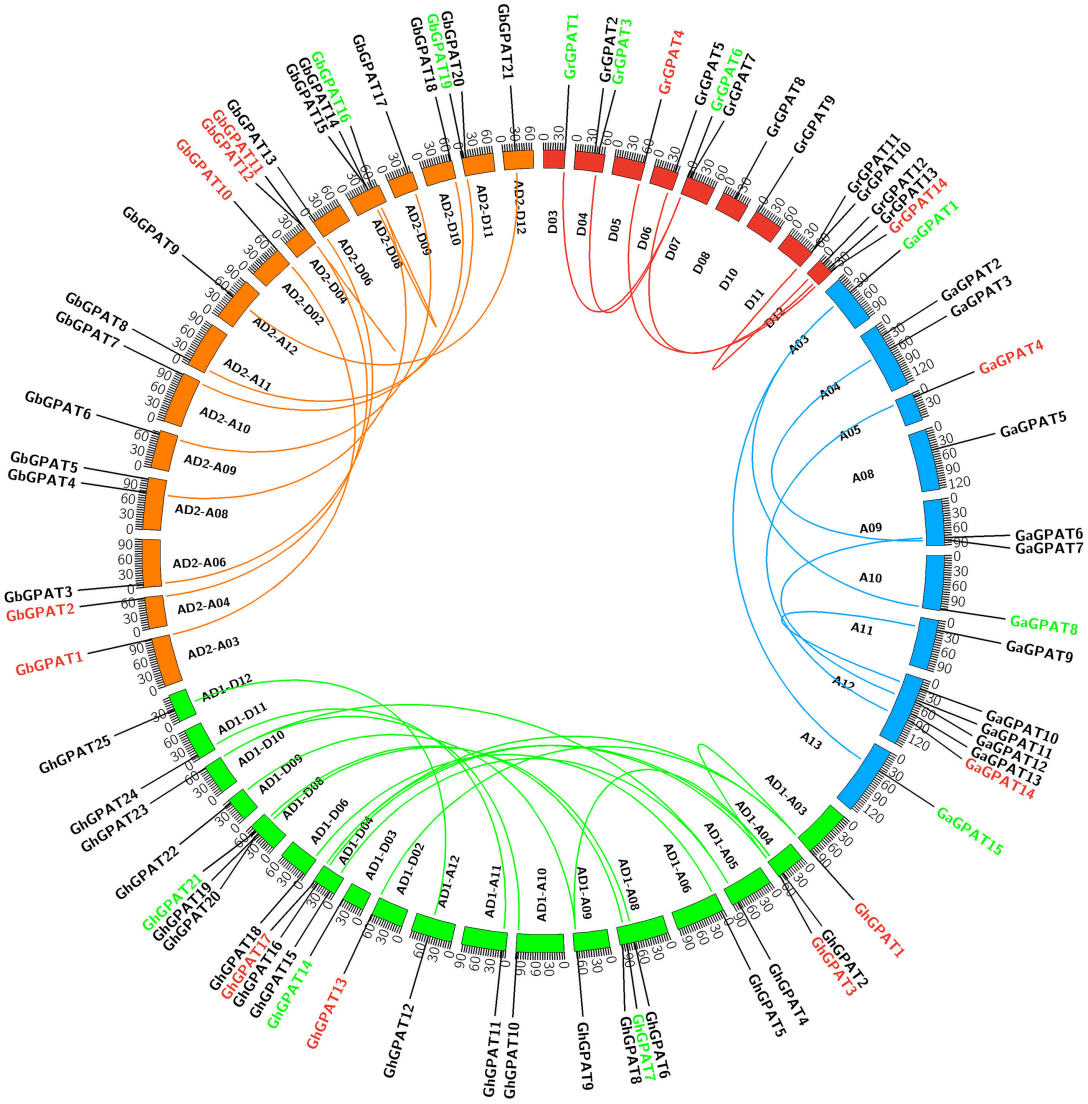
Genomic localization and gene duplication of *GPAT* genes in the four cotton species. The chromosomes of *G. raimondii, G. arboreum, G. hirsutum*, and *G. barbadense* were filled with red, blue, green, and orange, respectively. The duplicated gene pairs were connected with different color lines. Green, red, and black *GPAT* genes belonged to Group I, Group II, and Group III, respectively.

*GPAT* genes were mapped onto chromosomes in *G. arboreum, G. raimondii, G. hirsutum*, and *G. barbadense* based on the available genomic information on the four cotton species. Seventy-six of 85 *GPATs* were assigned to chromosomes (Figure 3), while the remaining 9 *GPATs* were anchored on unmapped scaffolds. A total of 14 *GrGPAT* genes were mapped to 9 chromosomes of *G. raimondii*: chromosome D12 contained three *GPAT* genes, chromosomes D04, D07, and D11 contained two *GPAT* genes each, and chromosomes D03, D05, D06, D08, and D10 each contained only one *GPAT* gene. The 16 *GaGPAT* genes were distributed on nine chromosomes in *G. arboreum*: 5 *GPAT* genes were on chromosome A12 and 2 genes on each of chromosomes A04, A09, and A13, while each of chromosomes A03, A05, A08, A10, and A11 contained only one *GPAT* gene. A total of 25 *GhGPAT* genes were mapped to 18 chromosomes of the *G. hirsutum* genome, not including chromosomes A01, A02, A07, A13, D01, D05, D07, and D13. The remaining three genes, *GhGPAT26, GhGPAT27*, and *GhGPAT28*, were positioned on unmapped scaffolds. As illustrated in Figure 3, chromosomes A08, D04, and D08 contained three *GPAT* genes each, and two *GPAT* genes were present on chromosome A04, while only one *GPAT* gene appeared on each of chromosomes A03, A05, A06, A09, A10, A11, A12, D02, D03, D06, D09, D10, D11, and D12. A total of 21 *GbGPAT* genes were distributed unevenly on 16 of 26 *G. barbadense* chromosomes, and the number of *GPAT* genes on each of the 16 chromosomes ranged from 1 to 3.

Gene duplication events were considered to play an important role in the amplification of gene families. It is reported that the genomes of *G. raimondii* and *G. arboreum* have undergone at least two rounds of genome-wide duplication (Paterson et al., 2012; Wang et al., 2012). And all tetraploid cotton species are derived from *G. raimondii* and *G. arboreum* (Wendel et al., 2009). Therefore, evolutionary relationships among the four cotton species are close. To elucidate the expanded mechanism of the *GPAT* gene family, gene duplication event analysis in the four cotton species was performed, and the details of the duplicated gene pairs were listed in Table 2. A total of 5, 6, and 19 duplication events were detected in *G. raimondii, G. arboreum*, and *G. hirsutum*, respectively. They were located on individual chromosomes and were mainly attributed to segmental duplication events in the three cotton species (Figure 3). In *G. barbadense*, 1 pair of tandem duplications and 13 pairs of segmental duplication events were detected (Figure 3, Table 2), but we also concluded that the expansion of the *GbGPAT* gene family occurred mainly by segmental duplication events rather than tandem events.

**Table 2:** Ka/Ks analysis for the duplicated *GPAT* gene pairs from *G. raimondii, G. arboreum, G. hirsutum*, and *G. barbadense*.

**Species**	**Duplicated gene 1**	**Duplicated gene 2**	**Ka**	**Ks**	**Ka/Ks**	**Purifying selection**	**Duplicate type**
*G. raimondii*	*GrGPAT1*	*GrGPAT6*	0.149	0.179	0.832	Yes	Segmental
	*GrGPAT4*	*GrGPAT14*	0.12	0.128	0.938	Yes	Segmental
	*GrGPAT5*	*GrGPAT13*	0.132	0.104	1.269	No	Segmental
	*GrGPAT6*	*GrGPAT3*	0.158	0.137	1.153	No	Segmental
	*GrGPAT10*	*GrGPAT12*	0.211	0.188	1.122	No	Segmental
*G. arboreum*	*GaGPAT1*	*GaGPAT8*	0.131	0.197	0.665	Yes	Segmental
	*GaGPAT1*	*GaGPAT15*	0.119	0.248	0.48	Yes	Segmental
	*GaGPAT3*	*GaGPAT7*	0.205	1.024	0.2	Yes	Segmental
	*GaGPAT4*	*GaGPAT14*	0.094	0.228	0.412	Yes	Segmental
	*GaGPAT6*	*GaGPAT12*	0.14	0.495	0.283	Yes	Segmental
	*GaGPAT9*	*GaGPAT10*	0.08	0.374	0.214	Yes	Segmental
*G. hirsutum*	*GhGPAT1*	*GhGPAT13*	0.007	0.008	0.875	Yes	Segmental
	*GhGPAT2*	*GhGPAT9*	0.11	0.163	0.675	Yes	Segmental
	*GhGPAT2*	*GhGPAT16*	0.007	0.008	0.875	Yes	Segmental
	*GhGPAT3*	*GhGPAT1*	0.089	0.115	0.774	Yes	Segmental
	*GhGPAT3*	*GhGPAT17*	0.009	0.008	1.125	No	Segmental
	*GhGPAT4*	*GhGPAT15*	0.005	0.016	0.313	Yes	Segmental
	*GhGPAT4*	*GhGPAT23*	0.174	0.311	0.559	Yes	Segmental
	*GhGPAT5*	*GhGPAT18*	0.01	0.033	0.303	Yes	Segmental
	*GhGPAT6*	*GhGPAT19*	0.017	0.016	1.063	No	Segmental
	*GhGPAT7*	*GhGPAT21*	0	0.008	0	Yes	Segmental
	*GhGPAT7*	*GhGPAT27*	0.122	0.174	0.701	Yes	Segmental
	*GhGPAT9*	*GhGPAT22*	0.005	0.016	0.313	Yes	Segmental
	*GhGPAT10*	*GhGPAT23*	0.03	0.036	0.833	Yes	Segmental
	*GhGPAT11*	*GhGPAT24*	0.01	0.007	1.429	No	Segmental
	*GhGPAT12*	*GhGPAT25*	0.016	0.018	0.889	Yes	Segmental
	*GhGPAT14*	*GhGPAT26*	0.009	0.026	0.346	Yes	Segmental
	*GhGPAT17*	*GhGPAT1*	0.084	0.125	0.672	Yes	Segmental
	*GhGPAT21*	*GhGPAT28*	0.126	0.15	0.84	Yes	Segmental
	*GhGPAT27*	*GhGPAT28*	0.017	0.016	1.063	No	Segmental
*G. barbadense*	*GbGPAT1*	*GbGPAT10*	0.006	0.008	0.75	Yes	Segmental
	*GbGPAT2*	*GbGPAT11*	0.008	0.015	0.533	Yes	Segmental
	*GbGPAT3*	*GbGPAT13*	0.014	0.008	1.75	No	Segmental
	*GbGPAT4*	*GbGPAT15*	1.812	1.535	1.18	No	Segmental
	*GbGPAT6*	*GbGPAT17*	0.011	0.015	0.733	Yes	Segmental
	*GbGPAT7*	*GbGPAT18*	0.032	0.059	0.542	Yes	Segmental
	*GbGPAT7*	*GbGPAT24*	0.234	0.178	1.315	No	Segmental
	*GbGPAT8*	*GbGPAT20*	1.705	3.103	0.549	Yes	Segmental
	*GbGPAT9*	*GbGPAT21*	0.006	0.016	0.375	Yes	Segmental
	*GbGPAT11*	*GbGPAT12*	0.006	0.015	0.4	Yes	Tandem
	*GbGPAT14*	*GbGPAT15*	1.889	1.525	1.239	No	Segmental
	*GbGPAT16*	*GbGPAT22*	1.876	1.557	1.205	No	Segmental
	*GbGPAT25*	*GbGPAT23*	0.008	0.024	0.333	Yes	Segmental
	*GbGPAT26*	*GbGPAT27*	0.01	0.007	1.429	No	Segmental

The duplicated gene pairs might have undergone three alternative fates during their evolution, i.e., nonfunctionalization, neofunctionalization and subfunctionalization (Lynch and Conery, 2000). To examine the selective constrains on duplicated *GPAT* gene pairs in the four cotton species, the Ka/Ks ratio for each pair of duplicated *GPAT* genes was calculated using the full-length sequences. Generally, Ka/Ks > 1 indicates accelerated evolution with positive selection, Ka/Ks = 1 indicates neuteal selection, while Ka/Ks < 1 indicates the functional constraint with purifying selection. In this study, there are 2, 6, 15, and 8 pairs of duplicated *GPATs* with a Ka/Ks ratio < 1 in *G. raimondii, G. arboreum, G. hirsutum*, and *G. barbadense*, respectively, which suggested that they had experienced purifying selection pressure with limited functional divergence after cotton *GPAT* genes duplications (Table 2). However, 3, 4, and 6 pairs of duplicated *GPATs* with a Ka/Ks ratio > 1 were found in *G. raimondii, G. hirsutum*, and *G. barbadense*, respectively, suggesting that these cotton *GPAT* genes had experienced positive selection (Table 2). These observations reflected that the functions of the duplicated *GPAT* genes in the four cotton species had not diverged much during subsequent evolution, and purifying selection might contribute greatly to the maintenance of function in the *GPAT* family genes in the four cotton species.

### Expression Profiling of GPAT Genes in Upland Cotton and Arabidopsis

Gene expression is the major step toward a gene realizing its biological function. To understand the temporal and spatial expression patterns of upland cotton and *Arabidopsis GPAT* genes, their expression profiles in different tissues were analyzed using the public expression data.

**Figure 4:**
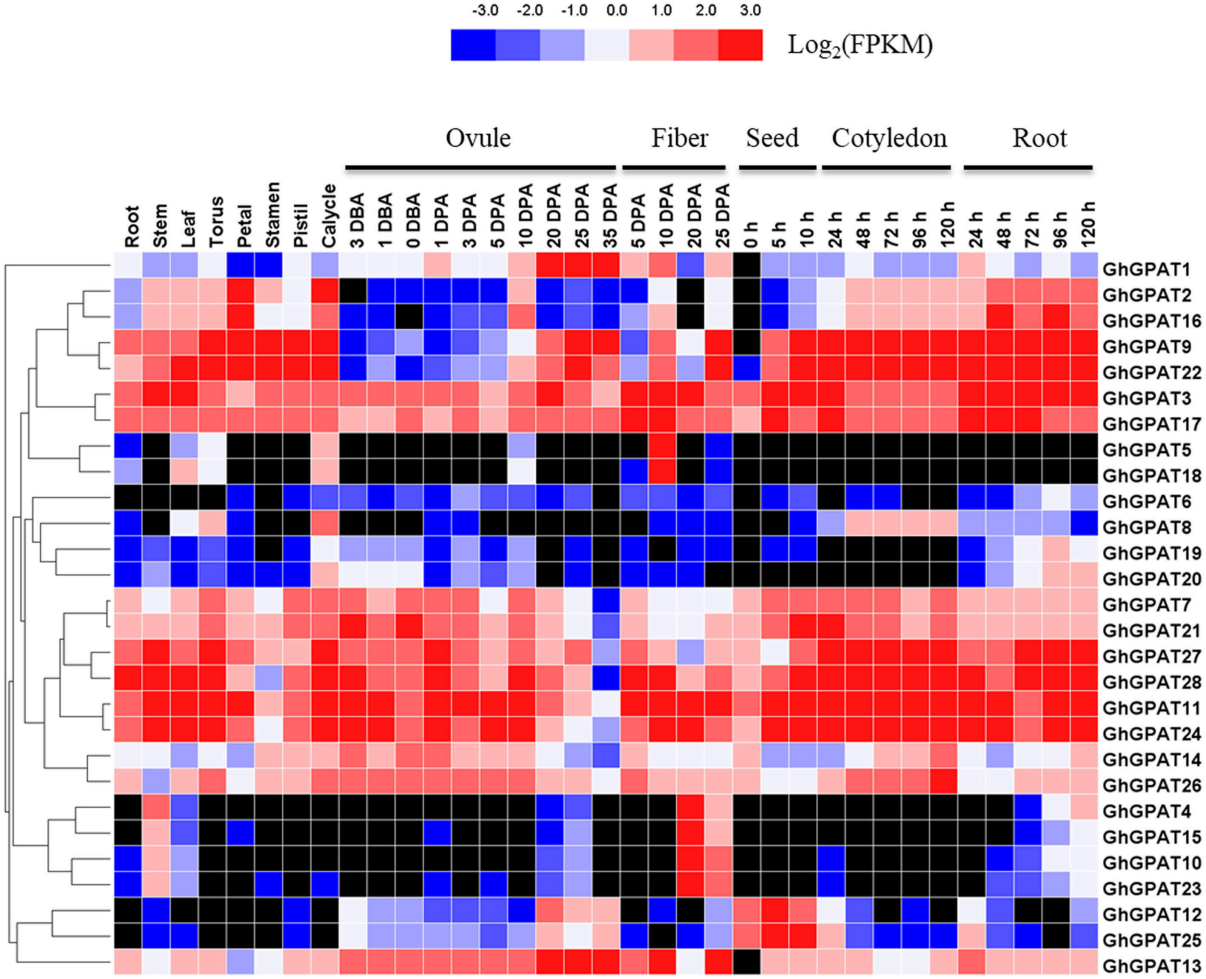
Expression profiles of *GhGPAT* genes in different tissues of *G. hirsutum* acc. TM-1. The RNA-Seq expression profiles of *G. hirsutum* acc. TM-1 (Zhang T. et al., 2015) were used to identify the expression levels of *GhGPAT* genes. The color bar represents the expression values in FPKM, and the black color represents there is not expression.

The RNA-seq data of *GhGPAT* genes from different tissues and developmental stages of *G. hirsutum* acc. TM-1 (Zhang T. et al., 2015) were analyzed (Figure 4). As shown in Figure 4, *GhGPAT* genes exhibited different expression patterns in different tissues of TM-1, indicating that *GhGPAT* genes had multiple biological functions during cotton growth and development. Eleven *GhGPAT* genes (*GhGPAT3, GhGPAT7, GhGPAT11, GhGPAT14, GhGPAT17, GhGPAT21, GhGPAT22, GhGPAT24, GhGPAT26, GhGPAT27*, and *GhGPAT28*) could be detected in all the tested tissues, indicating that these *GhGPAT* genes were involved in multiple processes during the development of cotton. With the exception of *GhGPAT5, GhGPAT6, GhGPAT8*, and *GhGPAT18*, the expression of other genes could be detected in the stems. *GhGPAT5* and *GhGPAT18* were specifically expressed in 10 DPA fibers, and *GhGPAT4, GhGPAT10, GhGPAT15*, and *GhGPAT23* were expressed in 20 DPA fibers, suggesting that these genes might play important roles in the rapid elongation period and secondary wall development. *GhGPAT13* exhibited a high level of accumulation during ovule development (−3 to 35 DPA) and fiber development (5, 10, and 25 DPA), indicating that *GhGPAT13* might play important roles in ovule development and fiber development. *GhGPA4, GhGPAT5, GhGPAT15, GhGPAT18, GhGPAT19* and *GhGPAT20* were not detectable at the different stages of cotyledons, suggesting that those six genes might not be associated with seed germination. There were 13 pairs of paralogous genes among these *GhGPAT* genes, and 11 pairs clustered together, which suggested that the expression of the paralogous *GhGPAT* genes might be conserved. In addition, the expression patterns of duplicated gene pairs in *G. hirsutum* were also investigated in Figure 4. Most duplicated gene pairs such as *GhGPAT2/GhGPAT16* and *GhGPAT5/GhGPAT18* were similar. However, the expression of *GhGPAT1* and *GhGPAT13* were strongly divergent, which might be caused by the significant variation in gene regulations after the duplication events.

The expression profiles of *Arabidopsis GPAT* genes were also analyzed unsing the public microarray data (Toufighi et al., 2005). Similar to *GhGPAT* genes, the *AtGPAT* genes also showed different expression patterns in different tissues (Figure S2). The expression of *AtGPAT5* showed a relatively high level in the siliques at 6–10 DAF (day after flowering), but lower expression in early developmental stages of silique (4–5 DFA), which suggested that it might contribute to the accumulation of glycerolipid in *Arabidopsis* siliques. The *AtGPAT6* had high exprsssion in sepals, petals and stamens than in other tissues, which indicated that it may play important rols in the *Arabidopsis* flower development. Additionally, the paralogous gene pairs of *Arabidopsis GPAT* genes were also investigated, it was showed that *AtGPAT4/AtGPAT8* had the simiar expression patterns. However, it was not the case for *AtGPAT2/AtGPAT3*.

### Gene Expression Patterns Under Salt and Cold Stresses

**Figure 5:**
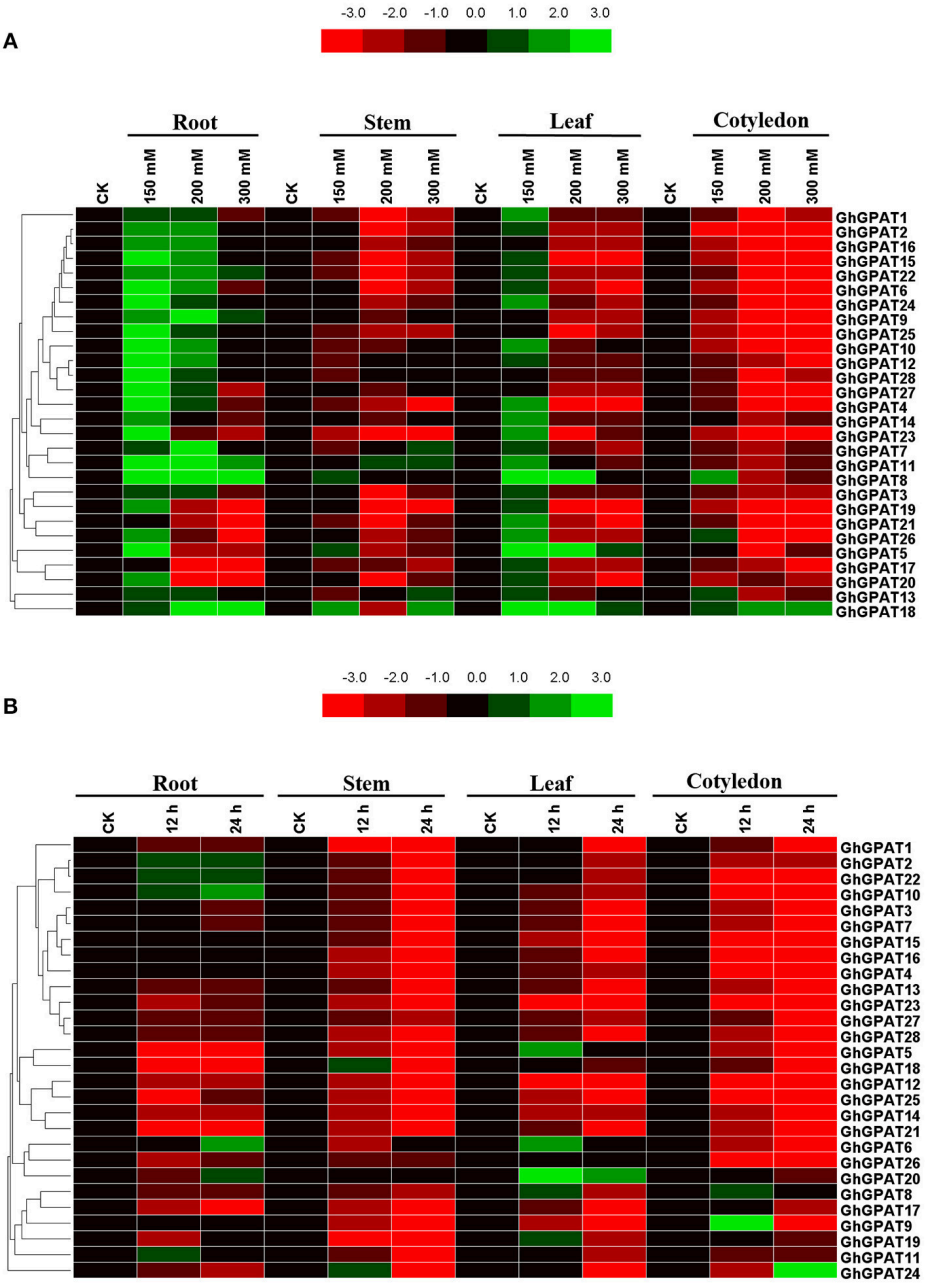
Expression patterns of 28 *GhGPAT* genes in four representative tissues of *G. hirsutum* in response to salt stress and cold stress. **(A)** Expression patterns of 28 *GhGPAT* genes under salt stress. **(B)** Expression patterns of 28 *GhGPAT* genes under cold stress. Slight stress, moderate stress, and severe stress in *G. hirsutum* were represented by 150, 200, and 300 mM NaCl, respectively. The color bar represents the relative signal intensity values.

Salt and cold stresses are the two of the main abiotic stresses that limit the productivity and geographical distribution of various crops all over the world. Previous study reported that plant *GPAT* genes are well known to be involved in responses to salt (Sun et al., 2010; Sui et al., 2017) and cold stresses (Murata and Tasaka, 1992; Ariizumi et al., 2002; Sui et al., 2007b), but the function of *GhGPAT* genes in cotton response to salt or cold stress remains unknown. The *cis*-elements could provide critical evidence and understanding about the functions of *GPAT* genes in cotton. Therefore, the promoter regions up to 2 kb upstream of all 28 *GhGPAT* genes were used to find their putative *cis*-elements (Table S4), and eight putative environmental stimulus-responsive *cis*-elements were identified in *GhGPAT* genes (Table S5). Although there were no *cis*-elements responding to salt stress and only one *cis*-element responding to low temperature in the PLACE database, some *cis*-elements might respond to multiple environmental stimuli (Higo et al., 1999). The results showed that almost all *GhGPAT* promoters contained more than three environmental stimulus-responsive *cis*-elements except for *GhGPAT13* (Table S5), indicating that *GhGPAT* genes may be involved in the response to salt and cold stresses.To validate the participation of *GPAT* genes in salt or cold stress, gene expression of all *GhGPAT* genes were conducted in different tissues under salt or cold stress (Figure 5). For the salt stress, the relative expression levels of *GhGPAT* genes were altered either induction or suppression under different salt treatments (Figure 5A). In the roots, nearly all *GhGPAT* genes were upregulated under slight salt stress except for *GhGPAT17* and *GhGPAT21*. Most *GhGPAT* genes were suppressed under severe stress except for *GhGPAT8, GhGPAT9, GhGPAT11, GhGPAT18*, and *GhGPAT22*. In the stems, most *GhGPAT* genes displayed downregulation under different levels of salt stress, while five *GhGPAT* genes (*GhGPAT5, GhGPAT7, GhGPAT8, GhGPAT11, GhGPAT13*, and *GhGPAT18*) showed upregulation under at least one salt treatment. In the leaves, *GhGPAT5* and *GhGPAT18* were upregulated under different levels of salt stress. Several *GhGPAT* genes showed downregulation. In the cotyledons, only *GhGPAT18* was upregulation under different levels of salt stress, while nearly all *GhGPAT* genes were downregulation. Furthermore, two duplicated gene pairs, *GhGPAT2/GhGPAT16* and *GhGPAT27/GhGPAT28*, showed similar expression patterns. For the cold stress (Figure 5B), only a few *GhGPAT* genes were induction, while others was suppression under cold stress. In the roots, five *GhGPAT* genes (*GhGPAT2, GhGPAT6, GhGPAT10, GhGPAT20*, and *GhGPAT22*) displayed upregulated expression under cold stress, while others were suppressed by cold stress. In the stems, *GhGPAT18* and *GhGPAT24* were initially increased at 12 h of cold stress and then decreased at 24 h of cold stress. In the leaves, the expression of *GhGPAT20* showed upregulation under cold stress as compared with other *GhGPAT* genes. In the cotyledons, most of the *GhGPAT* genes were suppressed under cold stress except for *GhGPAT8, GhGPAT9*, and *GhGPAT24. GhGPAT8* and *GhGPAT9* were upregulated after 12 h of cold treatment, while *GhGPAT24* was upregulated at 24 h. In addition, three duplicated gene pairs, *GhGPAT5/GhGPAT18, GhGPAT12/GhGPAT25*, and *GhGPAT27/GhGPAT28* were clustered together with similar expression patterns.

### Co-localization and Sequence Variation of GhGPAT Genes With Quantitative Trait Loci for Seed Oil and Protein Contents

**Figure 6:**
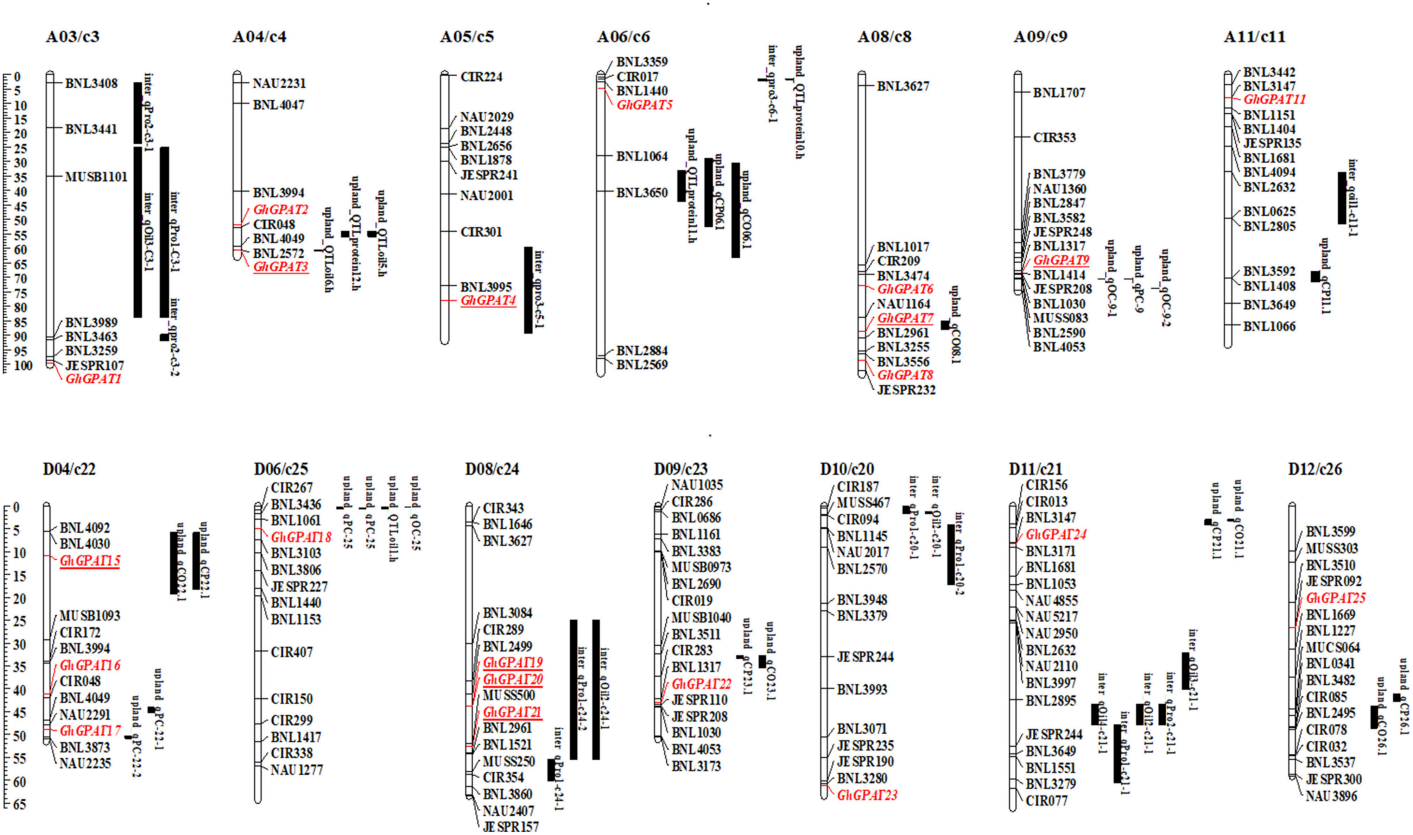
A co-localization analysis of *GhGPAT* genes with cotton oil and protein quantitative trait loci (QTLs). *GhGPAT* genes are shown in red, and underlining indicates the *GhGPAT* genes colocalized with oil or protein content QTLs.

To analyze the relationships of *GhGPAT* genes with cottonseed oil and protein contents, a co-localization analysis of *GhGPAT* genes with oil- and protein-related QTLs was conducted (Figure 6). QTLs for oil and protein traits were downloaded from intraspecific *G. hirsutum* and interspecific *G. hirsutum* × *G. barbadense* populations (Said et al., 2015a). The results showed that 5 *GhGPAT* genes on five chromosomes were co-localized within a 21.7 cM region of cottonseed oil- and protein-related QTLs, including *GhGPAT3* on chromosome A04/c4, *GhGPAT4* on chromosome A05/c5, *GhGPAT7* on chromosome A08/c8, *GhGPAT9* on chromosome A09/c9, and *GhGPAT15* on chromosome D04/c22. Three *GhGPAT* genes on one chromosome (*GhGPAT19, GhGPAT20*, and *GhGPAT21* on chromosome D08/c24) were localized within a 35 cM region (Figure 6). The co-localized QTLs include six QTLs (on chromosomes A04, A08, A09 and D04) from intraspecific *G. hirsutum* population and three QTLs (on chromosomes A05 and D08) from interspecific *G. hirsutum* × *G. barbadense* population (Table S6). In intraspecific *G. hirsutum* population, 4 and 2 QTLs associated with *GhGPAT* genes were identified for cotton oil and protein, respectively. *GhGPAT3* and *GhGPAT7* each co-localized with one oil-related QTL, while *GhGPAT9* and *GhGPAT15* each co-localized with one oil-related QTL and one protein-related QTL. In addition, one oil-related QTL and two protein-related QTLs associated with *GhGPAT* genes were identified from interspecific *G. hirsutum* × *G. barbadense* population. *GhGPAT4* only co-localized only with one oil-related QTL on chromosome A05, whereas *GhGPAT19, GhGPAT20*, and *GhGPAT21* co-localized with one oil-related QTL and one protein-related QTL on chromosome D08. However, the causative relationship between the natural variation in *GhGPAT* genes and oil content may not be obtained from the co-localization of a cottonseed oil-related QTL or a protein-related QTL with *GhGPAT* genes, since hundreds of genes could be identified from a 20 cM region (Said et al., 2015b), even 35 cM region.

To analyze the relationship between the sequence variation in *GPAT* genes and oil content, the SNPs of *GPAT* genes were identified from RNA-seq data on CRI 36 and Hai 7124. As shown in Table S7, 32 SNPs in the cDNA sequences of 12 *GPAT* genes were identified from the CRI 36 and Hai 7124 data compared with the TM-1 genome. Among them, 16 SNPs in 9 *GPAT* genes were classified as synonymous, and 16 SNPs in 10 *GPAT* genes were non-synonymous. To detect the association analysis between SNPs (non-synonynous) from *GPAT* genes and oil content, primers were designed to amplify fragments containing SNPs (Table S2) and analyzed using high-resolution melting (HRM). The 16 SNPs classified as non-synonymous were screened in a BIL population of 180 lines derived from a backcross between CRI 36 and Hai 7124, and correlation analysis was conducted in the BIL population of 180 lines in 2015 AY, 2016AY, and 2016XJ. An SNP of *GhGPAT16*, named *GhGPAT16*-1624 (T/C), which changes amino acid 542 from phenylalanine to leucine, was found to be significantly associated with oil content in the BIL population in 2015AY, and an SNP of *GhGPAT26*, named *GhGPAT26*-172 (T/C), which changes amino acid 58 from serine to proline, was found to be associated with oil content in 2016AY (Table S8). In the BIL population, the correlation of *GhGPAT16*-1624 and oil content in 2015AY was significantly negative (−0.262, *P* < 0.01), and the correlation of *GhGPAT26*-172 with oil content in 2016AY was significantly positive (0.183, *P* < 0.05). However, *GhGPAT16*-1624 and *GhGPAT26*-172 did not exhibit any association with oil content from the same BIL population in 2016AY, 2016XJ and 2015AY, 2016XJ, respectively. Therefore, the association between the SNP markers of *GhGPAT16* and *GhGPAT26* genes and oil content remains to be elucidated. These results suggest that the sequence variations in most *GhGPAT* genes are not associated with natural variations in cotton oil content.

## Discussion

### Identification, Phylogenetic Analysis and Evolution of GPAT Genes in Cotton

In this study, a total of 14, 16, 28, and 27 *GPAT* genes were identified from *G. raimondii, G. arboreum, G. hirsutum*, and *G. barbadense*, respectively (Table 1). The allotetraploid cotton species originated from an interspecific hybridization event between the progenitors *G. raimondii* (D5) and *G. arboreum* (A2) (Cronn et al., 1999; Wendel et al., 2009). Each of the *GPAT* genes in the diploid cotton species corresponds to two orthologous genes in their allotetraploid derivatives based on the theory of evolution. However, the numbers of *GPAT* genes in each of *G. hirsutum* and *G. barbadense* were slightly less than the sum of those from *G. raimondii* and *G. arboreum*, suggesting that whole-genome duplication was the major impetus and was followed by different degrees of gene loss during the evolution of allotetraploid cottons, and this was consistent with previous reported that gene loss was accompanied by the rapid arrangement of genomic sequences after hybridization and chromosome doubling during polyploidization (Paterson et al., 2004). This inconsistency has also been detected in a certain genus among different cotton species (Dong et al., 2017; Li et al., 2017; Pant et al., 2018). The number of *GPAT* genes in *G. arboreum* is slightly greater than that in *G. raimondii*, although the genome of *G arboreum* was approximately twice larger than that of *G. raimondii*, and the long terminal repeat (LTR) retrotransposon insertion in *G. arboreum* may explain the phenomenon (Paterson et al., 2012; Li et al., 2014). The *GPAT* family genes from the subgenomes of *G. hirsutum* or *G. barbadense* were more closely phylogenetically related to those from *G. raimondii* and *G. arboreum*, reflecting that the *GPAT* gene family evolved before the formation of allotetraploid cotton species.

Phylogenetic analysis revealed that *GhGPAT* genes were more closely related to *TcGPATs* than to *AtGPATs* (Figure 1), this result was consistent with the evolutionary relationships among the four cotton species, *cacao* and *Arabidopsis*. The *GPAT* genes from cotton, *Arabidopsis* and cacao were distinctly classified into three groups: Group I, Group II, and Group III (Figure 1), and no additional groups were identified in the tree, which was similar to those in *Arabidopsis* (Nishida et al., 1993; Gidda et al., 2009) and other plants (Waschburger et al., 2018). The gene structure analysis of *Arabidopsis* and cotton *GPAT* genes also supported this classification (Figure 2B). *GPAT* genes within the same groups showed similar exon numbers, indicating that genes within the same group diverged from a common ancestor. In addition, previous study reported that at least one rice *GPAT* gene was clusted with *Arabidopsis GPAT* gene from a phylogenetic tree constructed with 8 *Arabidopsis* and 16 rice *GPAT* genes (Horan et al., 2005). Take those results together, it suggested that the functional differences of various *GPAT* groups from *Arabidopsis*, rice and cotton have been conserved over 100 million years (Mya).

### Duplication of GPAT Genes in Cotton

Previous studies reported that gene duplication was the major reason for amplification of the gene family, including tandem duplication, segmental duplication, transposition events and whole-genome duplication (Blanc and Wolfe, 2004; Flagel and Wendel, 2009). Segmental duplication and translocation have enabled plants to rapidly adapt to new environments (Fraser et al., 2005). In this study, we analyzed gene duplication events to further understand the expansion mechanism of *GPAT* genes in cotton. Forty-four duplicated gene pairs were identified in the four cotton species, including 5, 6, and 19 segmental duplicated pairs detected in *G. raimondii, G. arboreum*, and *G. hirsutum*, respectively, and 1 and 13 pairs of tandem and segmental duplication detected in *G. barbadense* (Figure 3, Table 2). The results suggested that the expansion of cotton *GPAT* genes was mainly due to segmental duplication events. The results from Table 2 showed that purifying selection predominated across the duplicated gene pairs in cotton. Purifying selection could eliminate deleterious loss-of-function mutations, enhance fixation and preserve the function of a new duplicated gene at both duplicated loci (Tanaka et al., 2009). After duplication, the coding regions can obtain new regulatory context by insertion or deletion of tissue-specific enhancers or repressors, causing spatial and temporal variations in expression patterns of duplicated genes (Yang et al., 2017). The expression patterns of *GhGPAT* genes in different tissues of TM-1, showed that most of *GhGPAT* duplicated gene pairs displayed similar expression patterns, indicating that their biological functions might not be diverged during cotton development and growth, but determining whether these genes had similar functions in different tested tissues would require further studies. However, some *GhGPAT* duplicated gene pairs also displayed differential expression patterns, and this case also presented in the expression pattern of *Arabidopsis GPAT* genes (Figure S2), indicating that the biological functions of the duplicated genes might have experienced divergence during the development and growth of cotton and *Arabidopsis*, which might enable these two plants to rapidly adapt to new environments. Previous studies also reported that some duplicated gene pairs in other gene families, such as the *WOX* and *YABBY* gene families, displayed different expression patterns (Yang et al., 2017, 2018). These results indicated that divergence of duplicated gene pairs is a pervasive evolutionary process after duplication.

### Functional Divergence of Cotton GPAT Genes Under Salt Stress and Cold Stress

Salt and cold stresses are the serious environmental stresses that limit the productivity and geographical distribution of various crops worldwide. Previous studies revealed that the overexpression of *GPAT* genes in plants could enhance their salt or cold tolerance (Murata and Tasaka, 1992; Sui et al., 2007a, 2017; Sun et al., 2010). Gene expression patterns can provide useful clues for understanding gene function (Cui et al., 2017). Figure 5A showed that a majority of *GhGPAT* genes were upregulated in roots under moderate salt stress. In contrast, only a few upregulated *GPAT* genes were found in leaves compared with roots. This result was consistent with the fact that roots were the first tissues directly responding to salt stress (Guo et al., 2001), and root and leaf tissues were distinctly different in structure and function (Campo et al., 2014). Some *GhGPAT* genes showed similar expression patterns in the same tissue under salt or cold stress, being either induced or suppressed (Figure 5). For example, *GhGPAT8* and *GhGPAT11* were induced in roots by salt stress, and *GhGPAT2* and *GhGPAT22* were also upregulated before 24 h under cold stress. These results indicated that those genes were co-expressed under salt or cold stress. However, some *GhGPAT* genes in the same tissue also presented different expression patterns under salt stress and under cold stress. For instance, *GhGPAT18* was induced in roots under different salt stress conditions, while it was suppressed before 24 h under cold stress. This difference indicated that these genes may perform diverse functions under salt or cold stress. Together, these results implied that the signaling network responding to abiotic stress in plants was complicated (Zhang L. et al., 2015). In addition, some *GhGPAT* gene pairs presented a high degree of functional divergence under salt. *GhGPAT5* was downregulated in roots after severe salt stress, while its duplicated gene, *GhGPAT18*, was upregulated. This evidence supported the assertion that the expression divergence between duplicated gene pairs was the first step in functional divergence, which could increase the likelihood of their retention in the genome (Zhang, 2003).

## Conclusions

In this study, a total of 85 *GPAT* genes based on the genome information of *G. raimondii, G. arboreum, G. hirsutum*, and *G. barbadense* were identified. The family could be divided into three groups based on the phylogenetic tree and gene structures. Segmental duplication events were the main contributors to *GPAT* gene family expansion in *G. raimondii, G. arboreum, G. hirsutum*, and *G. barbadense*. Moreover, duplicated gene pairs of *GhGPATs* in upland cotton might be experienced functional divergence, since their expression patterns were different in different tissues. some *GhGPAT* genes are likely to be involved in salt or cold response. Eight *GhGPAT* genes were co-localized with oil and protein content QTLs, and two SNP markers were genetically associated with cottonseed oil content in one interspecific BIL population (in one of three field tests). Our results provide a solid foundation for further understanding about involvement of cotton *GPAT* genes in the natural variation of cottonseed oil content, and also could provide the candidate genes for function studies.

## Author Contributions

JZ and JY conceived and designed the study. YC and JM performed the experiments and analyzed the data. NW and GL prepared the figures. WP, MW, and XL analyzed and interpreted the data. YC, JZ, and JY prepared the manuscript. JZ participated in the design of the experiments, wrote part of the manuscript, and performed a critical review for intellectual content. All authors have read, edited, and approved the current version of the manuscript.

### Conflict of Interest Statement

The authors declare that the research was conducted in the absence of any commercial or financial relationships that could be construed as a potential conflict of interest.

## Supplementary Material

Figure S1 Phylogenetic relationships of *GPAT* genes from *Gossypium, Arabidopsis*, and *cacao*. The phylogenetic analysis was performed by Minimum likelihood method with 1,000 replicates. The GPAT genes from *G. raimondii, G. arboreum, G. hirsutum, G. barbadense, Arabidopsis*, and cacao were marked with the red, yellow, black, blue, green, and magenta dots, respectively. And the branches of each group were indicated in a specific color.

Figure S2 Expression profiles of *Arabidopsis GPAT* genes in different tissues. The Arabidopsis transcriptome of GPAT genes were conducted using the BioArray Resource (BAR) Expression Browser (Toufighi et al., 2005). The color bar represents the relative signal intensity values.

Table S1 *GPAT* genes of *Arabidopsis*, and *cacao* used to construct phylogenetic trees in this study.

Table S2 Primers for qRT-PCR and single nucleotide polymorphisms in this study.

Table S3 Orthologous and paralogous *GPAT* genes in and between the four cotton species.

Table S4 The *cis*-elements in the promoter regions of *GhGPATs*.

Table S5 The *cis*-elements involved in salt and cold stresses response in the promoter regions of *GhGPATs*. _indicated no corresponding cis-element.

Table S6 Oil and protein QTLs.

Table S7 Identification of putative sequence variation (SNPs) by SOAPsnp in CRI 36 vs. Hai 7124 database.

Table S8 Association analysis between the markers (GhGPAT16-1624 and GhGPAT26-172 from *GhGPAT16* and *GhGPAT26* genes) and cottonseed oil content in a BIL population.
