# Supplementary figures and images for: Genome-Wide Identification, Sequence Variation, and Expression of the Glycerol-3-Phosphate Acyltransferase (GPAT) Gene Family in Gossypium
Source: Front Genet. 2019 Feb 20;10:116. doi: 10.3389/fgene.2019.00116 (PMC6391866; doi:10.3389/fgene.2019.00116)

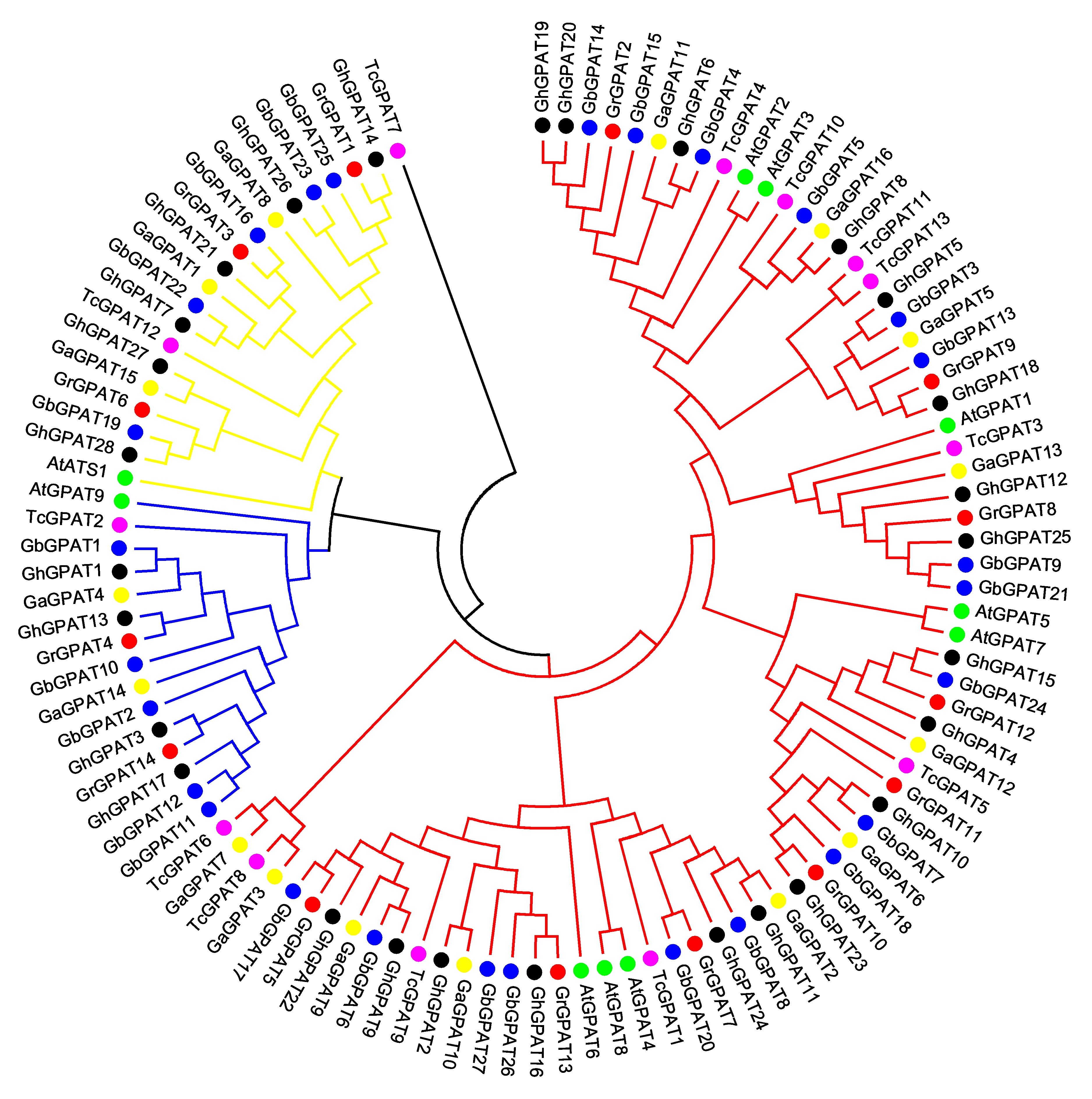

Supplement: Figure S1 — Phylogenetic relationships of GPAT genes from Gossypium, Arabidopsis, and cacao. The phylogenetic analysis was performed by Minimum likelihood method with 1,000 replicates. The GPAT genes from G. raimondii, G. arboreum, G. hirsutum, G. barbadense, Arabidopsis, and cacao were marked with the red, yellow, black, blue, green, and magenta dots, respectively. And the branches of each group were indicated in a specific color. [file Image_1.JPEG]

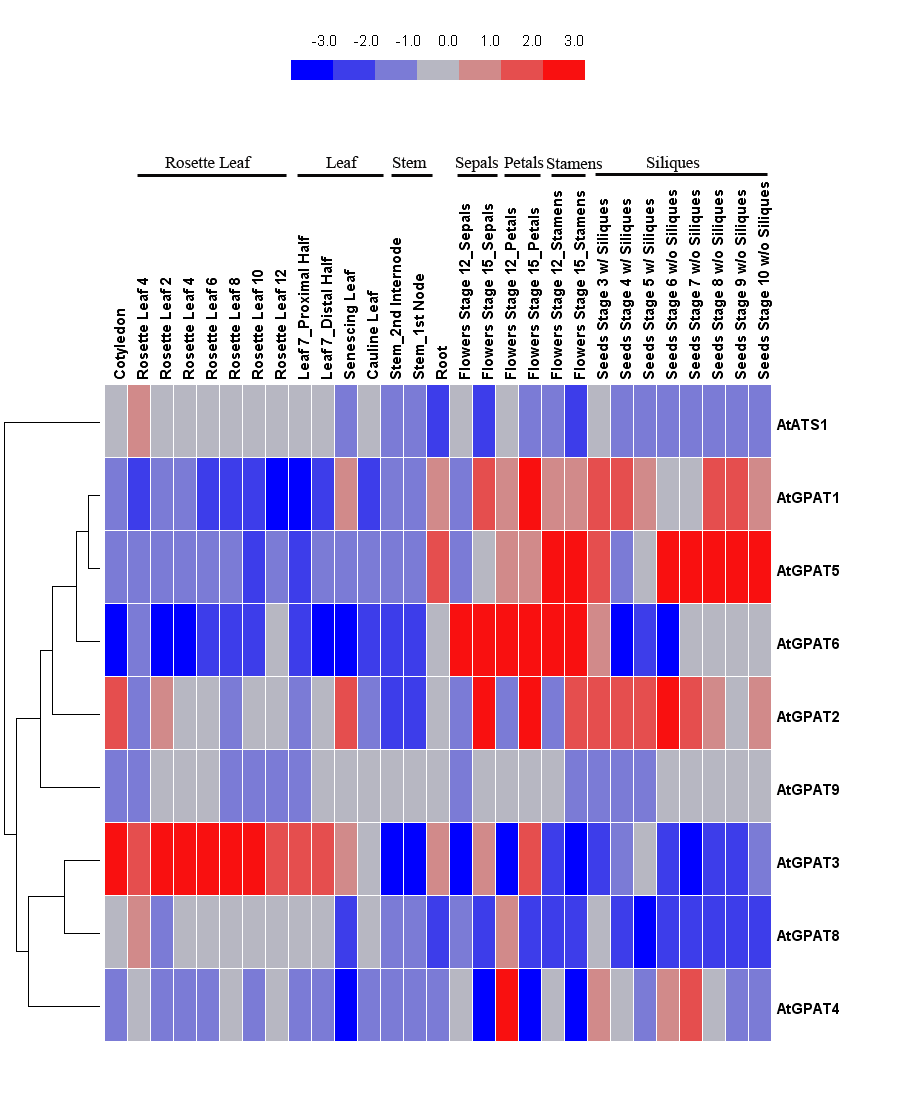

Supplement: Figure S2 — Expression profiles of Arabidopsis GPAT genes in different tissues. The Arabidopsis transcriptome of GPAT genes were conducted using the BioArray Resource (BAR) Expression Browser (Toufighi et al., 2005). The color bar represents the relative signal intensity values. [file Image_2.TIF]
