# Supplementary material for: Genome-Wide Identification, Sequence Variation, and Expression of the Glycerol-3-Phosphate Acyltransferase (GPAT) Gene Family in Gossypium
Source: Front Genet. 2019 Feb 20;10:116. doi: 10.3389/fgene.2019.00116 (PMC6391866; doi:10.3389/fgene.2019.00116)
Supplement: Table S1 — GPAT genes of Arabidopsis, and cacao used to construct phylogenetic trees in this study. [file Table_1.docx]

**Table S1. *GPAT* genes of *Arabidopsis*, and *cacao* used to construct phylogenetic trees in this study.**

| **Gene name** | **Accession NO.** | **Species** |
| --- | --- | --- |
| ***AtACT1*** | **AT1G32200** | ***Arabidopsis thaliana*** |
| ***AtGPAT1*** | **AT1G06520** | ***Arabidopsis thaliana*** |
| ***AtGPAT2*** | **AT1G02390** | ***Arabidopsis thaliana*** |
| ***AtGPAT3*** | **AT2G38110** | ***Arabidopsis thaliana*** |
| ***AtGPAT4*** | **AT4G01950** | ***Arabidopsis thaliana*** |
| ***AtGPAT5*** | **AT3G11430** | ***Arabidopsis thaliana*** |
| ***AtGPAT6*** | **AT1G01610** | ***Arabidopsis thaliana*** |
| ***AtGPAT7*** | **AT5G06090** | ***Arabidopsis thaliana*** |
| ***AtGPAT8*** | **AT4G00400** | ***Arabidopsis thaliana*** |
| ***AtGPAT9*** | **AT5G60620** | ***Arabidopsis thaliana*** |
| ***TcGPAT1*** | **XP_007051782** | ***Teobroma cacao*** |
| ***TcGPAT2*** | **XP_007041647** | ***Teobroma cacao*** |
| ***TcGPAT3*** | **XP_007048860** | ***Teobroma cacao*** |
| ***TcGPAT4*** | **XP_007051174** | ***Teobroma cacao*** |
| ***TcGPAT5*** | **XP_007009249** | ***Teobroma cacao*** |
| ***TcGPAT6*** | **XP_007044411** | ***Teobroma cacao*** |
| ***TcGPAT7*** | **XP_007032883** | ***Teobroma cacao*** |
| ***TcGPAT8*** | **XP_007032348** | ***Teobroma cacao*** |
| ***TcGPAT9*** | **XP_007026769** | ***Teobroma cacao*** |
| ***TcGPAT10*** | **XP_007031180** | ***Teobroma cacao*** |
| ***TcGPAT11*** | **XP_007018890** | ***Teobroma cacao*** |
| ***TcGPAT12*** | **XP_007040713** | ***Teobroma cacao*** |
| ***TcGPAT13*** | **XP_007018889** | ***Teobroma cacao*** |
