# Supplementary material for: Genome-Wide Identification, Sequence Variation, and Expression of the Glycerol-3-Phosphate Acyltransferase (GPAT) Gene Family in Gossypium
Source: Front Genet. 2019 Feb 20;10:116. doi: 10.3389/fgene.2019.00116 (PMC6391866; doi:10.3389/fgene.2019.00116)
Supplement: Table S2 — Primers for qRT-PCR and single nucleotide polymorphisms in this study. [file Table_2.docx]

**Table S2. Primers for qRT-PCR and single nucleotide polymorphisms in this study.**

| **Gene name** | **Forward primer (5'-3')** | **Reverse primer (5'-3')** |
| --- | --- | --- |
| ***GhGPAT1*** | **CCTGGATGGGTTGGACTACTGC** | **CCGATCTTTTGCCTCAGAACGGT** |
| ***GhGPAT2*** | **CACCTGCACCTAAGAAAGGCCA** | **TCGAGAAGGCGCTTGATGTCAG** |
| ***GhGPAT3*** | **AAGTACCATGGACCACGGCCTA** | **CGATCTTTGGCCTCTGAACGGT** |
| ***GhGPAT4*** | **ACTCGGTTCGGATTTGCTACGG** | **TTAAGAGAGCGGTCGAGGGTGT** |
| ***GhGPAT5*** | **ACCGGGCTGGCAAACACATAAT** | **CAGTTGCAATCTGACGCTGCAC** |
| ***GhGPAT6*** | **TGCCTTGTTGGCGAAGACATGA** | **TGGGCAAAACAGCACTCCCAAT** |
| ***GhGPAT7*** | **AGGAAGTAGATGCCGGAAGGCT** | **AACAGCTGCAGCATCTCCACTT** |
| ***GhGPAT8*** | **GGAACCACTTGCAGGGAACCAT** | **TATGGCAACCGGAACTATGCGG** |
| ***GhGPAT9*** | **ATCGAATCAGACAGTGGCTGCG** | **CCGACCCCGAAACAAGCTCAAT** |
| ***GhGPAT10*** | **GCGAATCATCCCCTATGTGACCC** | **AACGAATAGGACGCCGGAGTTG** |
| ***GhGPAT11*** | **TCAGCATCACCAGTACCGCTTG** | **GTGGATCCGGACGTTGGACAAA** |
| ***GhGPAT12*** | **GCAGGGCTGTTTTGCCAAAGTT** | **GTGCAATTCAGTGCCTGCAACA** |
| ***GhGPAT13*** | **ACTTGGTTGCACAATTTGCCCG** | **ATTCTGCGGCTCAAGGTACCAC** |
| ***GhGPAT14*** | **GTAGTGGAGGTGGAAGGGCAAC** | **TCTCGCAAAGCCTTGTGGTAGG** |
| ***GhGPAT15*** | **GGAAGCGACATGTTCATCGGGA** | **TCAAACCCTAACGTAGCAGCCA** |
| ***GhGPAT16*** | **GGTGCCAAGAACCAAATGCGAG** | **CTTAGGTGCAGGTGGAGGGGTA** |
| ***GhGPAT17*** | **GGCGTTGGTGGAGCTAATCTG** | **TTTCACGATCTTTGGCCTCTG** |
| ***GhGPAT18*** | **GGCATCCCTGCACCAACTCAAT** | **TCCACCAGCTTCAAAAGCTACCA** |
| ***GhGPAT19*** | **TTGTTGGCGAAGACATGGGGTT** | **ACAGCACTCCCAACCCTGAAAC** |
| ***GhGPAT20*** | **AAAGGATCTCACCGCCGTCAC** | **GCTCATCTCAGCGAACAAAGG** |
| ***GhGPAT21*** | **TTCCCAGAAATTGCTGCTGCCT** | **GACACCTGCAGTTGATGCCTCT** |
| ***GhGPAT22*** | **ATGGGGGCTTACCGTTGTTTCG** | **ATCTAAATCCGCAGCCACCGTC** |
| ***GhGPAT23*** | **CGAGCCGATGAGGTTGTTGGAA** | **ACGGGTAGTGGTCGAAGGAGTT** |
| ***GhGPAT24*** | **ACGTGTCGTGAGCAATTCCTGT** | **AATTTGACCCCTCTCACGGTCG** |
| ***GhGPAT25*** | **AAATCCTTGGAAAGGTGCCCCC** | **ACTCAAATCCCAGTGCATCGGC** |
| ***GhGPAT26*** | **CGTTTAGGCGGCTAGAGCTGTT** | **TCCTTCCACCTCCACTACGACC** |
| ***GhGPAT27*** | **ATGGCTGTAGCACTTGACCTG** | **TCGGCAATATGTGGATTTGTT** |
| ***GhGPAT28*** | **GAGGCGGCAAGATGGACATTGA** | **TCAGCAGCAGCAGGATCTTCAC** |
| ***GhUBQ7*** | **GAAGGCATTCCACCTGACCAAC** | **CTTGACCTTCTTCTTCTTGTGCTTG** |
| ***GhGPAT3-1191*** | **CTTGACGAGCAGGATTCATTCA** | **ATGGCTAACCTCATAGGGAACA** |
| ***GhGPAT11-3451*** | **TACGTGTATGAAAATTTGGGATG** | **TACTGGTGATGCTGATTTGCT** |
| ***GhGPAT13-102*** | **CTCCGAATTGGACTTGGATCG** | **GATGGGTTGAGGGAGGAGTGA** |
| ***GhGPAT13-3340*** | **CTCGCCCTAGCCCCAAGCAT** | **GCATTACAGGGAAAATTAAACAT** |
| ***GhGPAT14-4745*** | **GGGCAGGCTAAAGATACATACG** | **GCTTGATGCCGCAAACCCTTGT** |
| ***GhGPAT16-1582*** | **GTGCCAAATTCCTTCGAGATG** | **AACGGAGCTGCAAGTGACAAA** |
| ***GhGPAT16-1624*** | **GTGCCAAATTCCTTCGAGATG** | **GGAACGGAGCTGCAAGTGAC** |
| ***GhGPAT22-1775*** | **CAAATCTGGCAAGTTGGTCCCG** | **TTTAGGAGCTGACAAGGTTATT** |
| ***GhGPAT26-172*** | **TAGGCGGCTAGAGCTGTTTCG** | **TGTCCTTCCACCTCCACTACG** |
| ***GhGPAT27-42*** | **CTGAGACAAAGCTATACCTGCAG** | **ATGTCCTCAAATCAGCCATACA** |
| ***GhGPAT28-122*** | **CTTGTTTTCCATGTATGGCAGAT** | **CTTTTCCTTTTGGGTGAGCAG** |
| ***GhGPAT28-3977*** | **ACAGTTTCACTCGCTGAAACG** | **TGCTTTGTCTCTCCCATTCTTAA** |
