# Supplementary material for: Genome-Wide Identification, Sequence Variation, and Expression of the Glycerol-3-Phosphate Acyltransferase (GPAT) Gene Family in Gossypium
Source: Front Genet. 2019 Feb 20;10:116. doi: 10.3389/fgene.2019.00116 (PMC6391866; doi:10.3389/fgene.2019.00116)
Supplement: Table S5 — The cis-elements involved in salt and cold stresses response in the promoter regions of GhGPATs. _indicated no corresponding cis-element. [file Table_5.docx]

**Table S5. The *cis*-elements involved in salt and cold stresses response in the promoter regions of *GhGPATs*.** _indicated no corresponding *cis*-element.

| Gene name | Environmental stress-related element | | | | | | | |
| --- | --- | --- | --- | --- | --- | --- | --- | --- |
|  | HSE | LTR | MBS | TC-rich repeats | ARE | Box-W1 | W box | WUN-motif |
| *GhGPAT1* | 1 | - | 1 | 1 | 1 | 1 | 1 | - |
| *GhGPAT2* | 1 | - | 1 | 1 | - | - | 1 | - |
| *GhGPAT3* | - | 1 | 1 | 1 | 1 | - | - | - |
| *GhGPAT4* | 1 | - | 1 | - | 1 | - | 1 | - |
| *GhGPAT5* | 1 | - | 1 | 1 | 1 | - | - | - |
| *GhGPAT6* | 1 | - | - | - | 1 | - | 1 | - |
| *GhGPAT7* | 1 | - | 1 | - | 1 | 1 | 1 | - |
| *GhGPAT8* | 1 | - | 1 | 1 | - | 1 | 1 | 1 |
| *GhGPAT9* | 1 | - | 1 | 1 | - | - | 1 | - |
| *GhGPAT10* | 1 | - | 1 | - | - | - | 1 | - |
| *GhGPAT11* | 1 | 1 |  | 1 | - | - | - | - |
| *GhGPAT12* | 1 | - | 1 | 1 | 1 | 1 | 1 | - |
| *GhGPAT13* | - | - | 1 | - | - | - | - | - |
| *GhGPAT14* | 1 | - | 1 | - | 1 | - | - | - |
| *GhGPAT15* | 1 | - | - | 1 | 1 | - | - | - |
| *GhGPAT16* | 1 | - | 1 | 1 | - | 1 | 1 | - |
| *GhGPAT17* | 1 | 1 | - | 1 | 1 | - | - | - |
| *GhGPAT18* | 1 | - | - | 1 | 1 | - | - | - |
| *GhGPAT19* | 1 | - | 1 | 1 | 1 | 1 | 1 | - |
| *GhGPAT20* | 1 | 1 | - | 1 | 1 | - | - | 1 |
| *GhGPAT21* | 1 | - | 1 | 1 | 1 | - | 1 | - |
| *GhGPAT22* | 1 | - | 1 | 1 | 1 | 1 | 1 | - |
| *GhGPAT23* | 1 | - | 1 | - | 1 | 1 | 1 | - |
| *GhGPAT24* | 1 | - | - | 1 | - | 1 | - | 1 |
| *GhGPAT25* | 1 | - | 1 | 1 | 1 | 1 | 1 | - |
| *GhGPAT26* | 1 | - | 1 | 1 | 1 | - | - | 1 |
| *GhGPAT27* | - | - | 1 | 1 | 1 | 1 | 1 | - |
| *GhGPAT28* | - | - | 1 | 1 | 1 | - | - | - |

HSE: cis-acting element involved in heat stress responsiveness

LTR: cis-acting element involved in low-temperature responsiveness

MBS: MYB binding site involved in drought-inducibility

TC-rich repeats: cis-acting element involved in defense and stress responsiveness

ARE: cis-acting regulatory element essential for the anaerobic induction

Box-W1: fungal elicitor responsive element

W box: wounding and pathogen respons

WUN-motif: wound-responsive element
