# Supplementary material for: Genome-Wide Identification, Sequence Variation, and Expression of the Glycerol-3-Phosphate Acyltransferase (GPAT) Gene Family in Gossypium
Source: Front Genet. 2019 Feb 20;10:116. doi: 10.3389/fgene.2019.00116 (PMC6391866; doi:10.3389/fgene.2019.00116)
Supplement: Table S8 — Association analysis between the markers (GhGPAT16-1624 and GhGPAT26-172 from GhGPAT16 and GhGPAT26 genes) and cottonseed oil content in a BIL population. [file Table_8.docx]

**Table S8. Association analysis between the markers (GhGPAT16-1624 and GhGPAT26-172 from *GhGPAT16* and *GhGPAT26* genes) and cottonseed oil content in a BIL^a^ population.**

| Trait | SNP maarkers | 2015AY^b^ | 2016AY | 2016XJ^c^ |
| --- | --- | --- | --- | --- |
| Seed oil content (%) | *GhGPAT16-1624* | -0.262** | -0.045 | -0.059 |
|  | *GhGPAT26-172* | 0.128 | 0.183* | 0.11 |

^a^  BIL population was developed from a cross between Upland cotton CRI 36 and *G.barbadense* Hai 7124 through one generation of backcrossing using CRI 36 as the recurrent parent followed by seven generations of selfing.

^b^ AY: Anyang

^c^ XJ: Xinjiang

* and ** indicate correlation at the 0.05 and 0.01 significant levels, respectively.
